# Supplementary material for: Unified down-stream analysis of crosslinking mass spectrometry results with pyXLMS
Source: Nat Commun. 2026 Sep 4;17:10508. doi: 10.1038/s41467-026-77407-1 (PMC13638994; doi:10.1038/s41467-026-77407-1)
Supplement: Supplementary file 3 [file 41467_2026_77407_MOESM3_ESM.pdf]

nature portfolio

Peer Review File

Unified down-stream analysis of crosslinking mass spectrometry results with pyXLMS

Corresponding Author: Mr Micha Birklbauer

This file contains all reviewer reports in order by version, followed by all author rebuttals in order by version.

Attachments originally included by the reviewers as part of their assessment can be found at the end of this file.

Version 0:

Reviewer comments:

Reviewer #1

(Remarks to the Author)
The manuscript "Unified down-stream analysis of crosslinking mass spectrometry results with pyXLMS" presents a useful and timely framework to make intuitive the analysis across different search engines. The description of the data processing is clear and informative, and the algorithm is said to speed up the post-analysis not only for bioinformaticians but also for non-bioinformaticians. However, important aspects of the post-analysis, interoperability, and implementation details need clarification or correction to match the claims made in the text and abstract.

1) Level of analysis: CSM/XL vs PPI
The manuscript repeatedly states that pyXLMS focuses on CSM and XL aggregation and analysis. However, current XLMS applications place strong emphasis on PPI level questions (interactomes, network perturbation, ligand or toxin targeting, etc.), and this is also how the method is motivated in the abstract and introduction.
• The current description does not clearly explain if or how CSM and XL information is systematically propagated to PPI level (e.g. how protein–protein interactions are defined, how ambiguous peptides are handled at PPI level, or how multi protein groups are treated).
• FDR control at PPI level is not described, whereas FDR is discussed only for CSM and XL levels and only for a subset of engines (MaxLynx, MS Annika, xiSearch).

In this context, I would suggest to:
a) explicitly describe how pyXLMS derives PPIs from XLs: rules for grouping crosslinked residue pairs into protein–protein interactions, how ambiguity is handled, and how intra vs inter protein links are converted into network edges.
b) clarify whether PPI level FDR is implemented, and if not, state this limitation clearly and discuss how users are expected to control error rates at PPI level (e.g. via external tools, recommended workflows, or future work).
c) If PPI output is not supported, temper statements in the abstract and introduction regarding “identification of protein–protein interactions” to make clear that pyXLMS currently operates at CSM/XL level and relies on downstream tools to infer PPIs.

2) mzIdentML adoption and positioning
The introduction claims that mzIdentML 1.2/1.3 has “not seen wide adoption in the field of XLMS with only a handful of crosslink search engines and down-stream analysis tools supporting the format.” At the same time, you list mzIdentML 1.2 support in pyXLMS and emphasize interoperability as a main contribution. Given that many current engines do export mzIdentML 1.2/1.3, this statement feels outdated and undersells the existing ecosystem.

In this regard please:
a) Re evaluate and soften the statement on “not seen wide adoption,” or back it up with an up to date, quantitative overview of which crosslink engines and viewers support mzIdentML 1.2/1.3.
b) Highlight that pyXLMS can act as a bridge layer on top of the emerging mzIdentML 1.3 crosslink support, rather than implying the standard is rarely used.
c) Consider recommending mzIdentML 1.2/1.3 as a default interchange format for XLMS, with pyXLMS adding convenience and export functions on top of it.

3) FDR and engine coverage
The manuscript states that FDR estimation in pyXLMS is "currently only the case for MaxLynx, MS Annika, and xiSearch," because these provide targets/decoys and a single score. However, Scout is also described as providing CSV output and is included among the supported engines, and you mention reading both CSM and XL levels for Scout.

In the current XLMS landscape, Scout can produce target/decoy information at CSM, XL, and PPI levels, and it would be important to clarify if and why this is (not) used in pyXLMS. If technical reasons prevent using Scout's outputs for FDR, explain the limitation explicitly (e.g. missing combined score, multiple scoring dimensions, or format specifics). In addition, discuss whether PPI level FDR is planned, in particular leveraging engines like Scout that provide decoy PPIs.

4) Intersection of multiple engines in the web app
The manuscript claims that pyXLMS "allows to easily create intersections of CSMs and XLs, either from different replicates or different crosslink search engines." In the web application description, users are guided through upload, filtering, visualization, and export.
In practice, when using the public web app, the upload step enforces selection of a single engine/format from a dropdown, making it unclear how intersections across different search engines can be created in the GUI workflow you advertise for non programmers.

In this context, I would suggest clarifying whether multi engine intersections are currently only available in the Python API, and if so, state this explicitly and adjust the claims about the web app accordingly. Additionally, provide a brief and explicit example (user interface steps or code) demonstrating how to obtain the intersections of XLs in pLink, Scout, and XlinkX for the Cas9 dataset.

5) Export options vs Table 2 (xiView, MS Annika)
Table 2 lists xiVIEW as a supported export format (XL only) and does not mention MS Annika as an export target. The text emphasizes "more than ten different down-stream analysis tools and formats" and cites xiVIEW as a primary example. In the public web app, however, the export options presented to the user include only IPM X FDR, MS Annika, ProXL and xiFDR, with xiVIEW not available and MS Annika not listed in Table 2.

So, I would suggest reconciling Table 2, the main text, and the web app implementation. Ensure that xiVIEW export is consistently available in the web app if claimed in the manuscript or explicitly mark it as "currently only via Python API" if that is the case. In addition, add a note in the Methods or Implementation section stating that the set of export formats in the hosted web app may lag behind the Python package and specify what is guaranteed at the time of publication.

6) Versioning and Scout choice for the benchmark
Section 4.1 states that you tested Scout version 1.6.3 (Borges repository) and Scout 2.0.0 beta (Liu Lab repository). In the data analysis section, you report using Scout 2.0.0 beta from the Liu Lab for the Cas9 benchmark. For many users, the stable Scout 1.6.3 (or 2.0.0) from the Borges repository is now a practical default and, in some datasets, performs better than the beta variant.
In this case, justify the exclusive use of the beta 2.0.0 version in the main analysis; at minimum, mention that alternative releases (e.g. Scout 2.0.0 from Borges) exist and might produce different or improved results.
Moreover, if feasible, re run the Cas9 dataset with the stable Scout 2.0.0 (Borges) and report CSM, XL, and PPI numbers at 1% FDR alongside the existing pLink and XlinkX results. If re analysis is not possible, explicitly discuss how sensitive your conclusions (e.g. relative performance of pLink vs Scout) might be to the specific Scout version used.

7) Installation requirements and reproducibility
The Implementation section details Python packages and testing (pandas, pyteomics, biopython, matplotlib, biopandas, lxml, streamlit, sphinx, etc.) and reports 584 tests covering parsing and core functionality. However, minimal system requirements are not stated explicitly.
However, there are a lack information about the package versions used during the development as well as the minimum set up for the machine. In this context, add a short subsection (e.g. "System requirements") specifying at least: minimum Python version (e.g. 3.9+), required operating systems (Linux, macOS, Windows) and tested versions, approximate RAM/disk requirements for typical XLMS datasets, and any known performance constraints.

On the other side, for the web app, clarify what is needed to run locally or self host (Python version, streamlit version, browser compatibility). Since the authors emphasized non expert usability, make sure the user guide (cited URLs) includes a minimal "one page" install recipe that matches the versions used in this manuscript.

Minor comments and suggestions
1. Consistency in engine and format lists
The text at multiple points counts "seven different crosslink search engines" but then lists MaxLynx, MeroX, MS Annika, pLink 2/3, Scout, xiSearch/xiFDR, and XlinkX, which reads as more than seven logical tools depending on how they are grouped. For clarity, define whether xiFDR and XlinkX are treated as separate input engines or as auxiliary formats.
2. Clarify the mzIdentML version support
The authors explicitly mention support for mzIdentML 1.2 in the tests, and cite mzIdentML 1.3 as a format that extends crosslink reporting. It would be helpful to state clearly whether 1.3 is fully supported as input (and tested) or treated as 1.2 with partial coverage.
3. Documenting limitations more prominently

The Discussion already notes that lack of decoys in some engines prevents validation and export to xiFDR and that varying information content across engines limits some exports. It would help readers if the web app and docs expose these limitations explicitly (e.g. when a user uploads MeroX results and tries to export to xiFDR, a clear message that FDR cannot be recomputed in pyXLMS).
4. Terminology
Ensure consistent use of terms like “crosslink/residue pair,” “XL,” and “PPI” throughout the text and figures and clarify early on that the core data structures operate at CSM and XL levels, with PPIs derived rather than directly stored.

(Remarks on code availability)
I would suggest to create unit_tests for the code. For instance, src/pyXLMS/exporter requires unit tests for each parser.

Another suggestion would be to call pre commit hook before committing the code to make the repository more clear.

Reviewer #2

(Remarks to the Author)
In this manuscript, the authors present their pyXLMS tool that could essentially re-format output from different crosslink search engines, compatible with different downstream analysis tools. This tool is helpful especially for experimental researchers by reformatting their XLMS data so that they can input that data to other published analysis tools. Though the authors claim that their tool can also perform data analysis, the features that constitute this claim are mainly for data summarization rather than significant data analytical capability. As described in detail in the comments below, this tool is of limited use for the field and not suitable for publication in Nature Communications:
(a) The manuscript initially focuses on how their tool allows data reformatting and summarization on an example dataset. Later, the authors input this reformatted data to multiple published tools such as xiVIEW, AlphaFold2, AlphaLink2 and report and compare the results. The entire Figure 3 is dedicated towards showing these results and the corresponding text sounds like a tutorial for using these other tools. Additionally, Figure 2 and 4 essentially show the visualization of the data summary, which could just be presented as a single figure. Finally, Figure 5 just shows a snapshot of the tool’s user guide, which does not qualify as a full figure.

(b) I tested the webserver (http://pyxlms.projekte.fh-hagenberg.at) using an example dataset from their repository (https://github.com/hgb-bin-proteomics/pyXLMS/blob/master/data/ms_annika/XLpeplib_Beveridge_QEx-HFX_DSS_R1_CSMs.txt). When I clicked run, it started running but threw the following error: “Something went wrong! This is most likely due to missing information in the results!
KeyError: 'st.session_state has no key "aggregated". Did you forget to initialize it? More info: https://docs.streamlit.io/develop/concepts/architecture/session-state#initialization'
Traceback:
File "/pyXLMS/gui/streamlit_app.py", line 642, in input_tab
if st.session_state["aggregated"] is not None:
~~~~~~~~~~~~~~~~^^^^^^^^^^^^^^
File "/usr/local/lib/python3.12/site-packages/streamlit/runtime/state/session_state_proxy.py", line 101, in __getitem__
return get_session_state()[key]
~~~~~~~~~~~~~~~~~~~^^^^^
File "/usr/local/lib/python3.12/site-packages/streamlit/runtime/state/safe_session_state.py", line 104, in __getitem__
return self._state[key]
~~~~~~~~~~~^^^^^
File "/usr/local/lib/python3.12/site-packages/streamlit/runtime/state/session_state.py", line 478, in __getitem__
raise KeyError(_missing_key_error_message(key))”
Looking at the error message, I guessed perhaps it was because I selected “Validate aggregated crosslinks via FDR estimation option” but did not select “Aggregate crosslink spectrum matches to crosslinks” option. Once I selected it too, the error was gone. The server needs to be thoroughly tested for such inconsistencies especially because it was aimed at helping the experimental researchers and we cannot expect them to trouble-shoot based on the trace-back error log.

(c) Question about the definition of interprotein crosslinks: For the example I tested, there was an “intraprotein” CSM with both peptides alpha and beta as “GQKNSR” and the position of that crosslink in protein is also identical (779), indicating that the crosslink occurred between two different molecules of Cas9 protein. In this case, shouldn’t it be classified as “interprotein”, since it is between to different protein molecules albeit the same id? Example for a different but similar case is crosslinks between different subunits of a homo-oligomeric protein.
(d) It would be helpful to the users if the crosslink level table has an additional column showing how many CSMs were aggregated to give that crosslink. Additionally, the “Completeness” column in the CSM table has “partial” for all entries and the crosslink table has “complete” for all the entries. Could the authors explain the difference? I tried to find an explanation for this column in the tutorial but was unable to find it.

(e) Page 5:“Moreover, pyXLMS is able to deduplicate and filter CSMs and XLs to only contain unique matches, something that is desired for almost all down-stream analyis methods”. Is it mandatory to remove redundancy for most of the downstream analysis methods? Can the authors provide examples of such methods?
~~~~~~~~~~~

(f) The authors list ‘validation’ of the data as one of the key functionalities of their tool but it mainly performs FDR estimation, and it is only applicable to data from a limited number of search engines as acknowledged by them (page 5: “However, validation is limited to results from crosslink search engines that report both target and decoy matches, as well as a single score for FDR estimation. This is currently only the case for MaxLynx, MS Annika, and xiSearch.”). Perhaps, implementing quality assessment metrics such as the fraction of interprotein crosslinks as an indicator of data quality (PMID: 31267744) and the overlap of interprotein crosslinks with known interactions from public repositories (PMIDs: 32994567, 37071682, 34349018) could add value towards assessing the data quality.

(g) Page 3: “We are also hosting a publicly available and free of charge web server that includes most functionality …”. Does “includes most functionality” mean the standalone tool has more functionalities compared to the web server?

(Remarks on code availability)

Reviewer #3

(Remarks to the Author)
I co-reviewed this manuscript with one of the reviewers who provided the listed reports. This is part of the Nature Communications initiative to facilitate training in peer review and to provide appropriate recognition for Early Career Researchers who co-review manuscripts.

(Remarks on code availability)

Reviewer #4

(Remarks to the Author)
The authors present a python package and associated web interface that is able to take input from 7 different crosslink search algorithms. It can perform several basic tasks, including summarizing the data, filtering the data, calculating summary statistics and re-annotating crosslinks in cases where the identified peptides match multiple proteins. Importantly, pyXLMS is also able to output the data in formats that can then be read by and imported into several popular crosslink data analysis tools.
While the tool does perform several potentially useful functions many are already offered by existing tools. I do appreciate the author's point that if a researcher's XL analysis algorithm does not produce output compatible with the analysis tool they wish to use downstream then they need to convert their data. It is the reviewers opinion that this package could be useful to researchers wanting to convert crosslink data formats from the native output of the algorithm they used into a format suitable for use as input to one of these downstream analysis tools and I appreciate the effort made to cater for a wide range of inputs and outputs. I cannot recommend this work to be published in Nature Communications as it is not of sufficient novelty or impact. I think this work would be more suitable for submission as a brief technical note to a proteomics-specific journal (JPR, JASMS or similar).

(Remarks on code availability)

Version 1:

Reviewer comments:

Reviewer #1

(Remarks to the Author)
Reviewer' comments:
Thanks for reviewing the manuscript. Below I point some comments out.

1) Level of analysis: CSM/XL vs PPI
The manuscript repeatedly states that pyXLMS focuses on CSM and XL aggregation and analysis.
However, current XLMS applications place strong emphasis on PPI level questions (interactomes, network perturbation, ligand or toxin targeting, etc.), and this is also how the method is motivated in the abstract and introduction.
• The current description does not clearly explain if or how CSM and XL information is systematically propagated to PPI level (e.g. how protein–protein interactions are defined, how ambiguous peptides are handled at PPI level, or how multi protein groups are treated).
• FDR control at PPI level is not described, whereas FDR is discussed only for CSM and XL levels and only for a subset of engines (MaxLynx, MS Annika, xiSearch).

In this context, I would suggest to:
a) explicitly describe how pyXLMS derives PPIs from XLs: rules for grouping crosslinked residue pairs into protein–protein interactions, how ambiguity is handled, and how intra vs inter protein links are converted into network edges.
b) clarify whether PPI level FDR is implemented, and if not, state this limitation clearly and discuss how users are expected

to control error rates at PPI level (e.g. via external tools, recommended workflows, or future work).
c) If PPI output is not supported, temper statements in the abstract and introduction regarding "identification of protein–protein interactions" to make clear that pyXLMS currently operates at CSM/XL level and relies on downstream tools to infer PPIs.
We apologize that this was not made clear enough in the manuscript, pyXLMS exclusively works on CSM and XL level and does not support PPIs directly. This decision was made because we currently do not support any down-stream analysis tools that would require PPIs as input (and have not encountered such a tool yet). Some of the down-stream analysis tools might output PPIs using the inputs generated by pyXLMS, but handling aggregation and validation is then happening on the side of the down-stream analysis tool. We might support PPI input in the future if we see enough use cases but currently have no plans to implement them.
We have gone through the abstract and introduction again and PPIs are only mentioned as being applications of XLMS and not in the context of pyXLMS, we apologize if that came across wrong. We have added a statement in the pyXLMS description and in the limitations that explicitly clarifies that pyXLMS does not support PPIs directly at the moment.
Actions taken:
Manuscript: We explicitly added that pyXLMS does not directly support PPIs as a limitation in both the pyXLMS description in the introduction, results, and in the discussion.
Software: We have added a FAQ (see https://pyxlms.dev/docs/faq) that specifically clarifies which aggregation levels pyXLMS supports and that PPIs are not supported.

Thank for the explanation. However, it is important to note that, in contemporary XL-MS applications, the primary biological motivation is almost invariably the mapping of protein–protein interactions (PPIs) and interaction networks, rather than the isolated reporting of crosslinked residue pairs. Recent reviews explicitly position XL-MS as a tool for PPI discovery and characterization and for constructing dynamic interactomes at the systems level. Representative studies describe XL-MS–based efforts to define protein interaction landscapes in vivo, to perform in situ interaction proteomics and map extensive PPI maps in human cells and chromatin, and to leverage XL-MS for understanding endogenous PPI networks in the context of drug development against PPI modulators. Further recent work emphasizes XL-MS as a powerful approach for capturing protein network dynamics, from targeted mapping of PPIs to large-scale identification of interactome networks in cells and tissues. These examples illustrate that the field now routinely frames XL-MS as a PPI-centric technology, supporting the suggestion that the manuscript should more explicitly address how CSM/XL results are intended to be used for PPI-level questions and error control. Below you can find out some references that support the points above mentioned:

• Dynamic Interactomics by Cross-Linking Mass Spectrometry: Mapping the Daily Cell Life in Postgenomic Era – OMICS 2022.
o Highlights XL-MS as a powerful tool for PPI discovery and characterization and for capturing dynamic interactomes.

• Chemical cross-linking and mass spectrometry enabled systems-level structural biology – Curr Opin Struct Biol 2024.
o Emphasizes XL-MS for understanding protein–protein interaction networks relevant to physiology, pathology, and therapeutic targeting.

• Protein interaction landscapes revealed by advanced in vivo cross-linking–mass spectrometry – PNAS 2021.
o Describes XL-MS applied to define protein interaction landscapes and PPIs in their native cellular environment.

• Optimized Cross-Linking Mass Spectrometry for in Situ Interaction Proteomics – J Proteome Res 2019.
o Presents an XL-MS workflow for in situ interaction proteomics, mapping extensive protein–protein interaction maps in live human cells and chromatin.

The authors have provided clear and satisfactory responses to the remaining points raised in my review, adequately addressing the concerns about data processing description and the intended user audience. In particular, their clarifications on implementation details and interoperability improve the transparency of the manuscript and bring it closer to the claims made in the abstract and introduction. However, it remains critically important to explicitly clarify the points raised regarding the level of analysis (CSM/XL vs PPI), as discussed in my previous comments. Given that the current XL-MS field overwhelmingly frames the technology as a PPI-centric tool for interactome and network analysis, leaving this aspect ambiguous risks misrepresenting the method's scope and limiting its interpretability for downstream users. Therefore, while I am satisfied with the authors' responses to the other questions, I strongly recommend that the revised manuscript include a clear statement on how (or whether) crosslinked residue pairs are propagated to PPI level, how ambiguity is handled at that level, and how error control is intended to operate for PPI-derived conclusions, or at least explicitly acknowledge these as limitations and outline a recommended workflow for users.

(Remarks on code availability)
The Python code provided adheres to the standard rules of Python syntax and semantics: it uses valid identifiers, correct indentation, proper punctuation (parentheses, brackets, and commas), and follows the language's statement and expression structure. All imports, function definitions, loops, conditionals, and data manipulations are written in a way that would execute without syntax errors in a standard Python environment. The logic is clear, the variable names are meaningful, and the code is organized in a readable, maintainable form, making it satisfactory for the intended purpose.

Reviewer #2

(Remarks to the Author)

(Remarks on code availability)

Reviewer #4

(Remarks to the Author)
My concerns have been rebutted in the revisions.

(Remarks on code availability)

Open Access This Peer Review File is licensed under a Creative Commons Attribution 4.0 International License, which permits use, sharing, adaptation, distribution and reproduction in any medium or format, as long as you give appropriate credit to the original author(s) and the source, provide a link to the Creative Commons license, and indicate if changes were made.
In cases where reviewers are anonymous, credit should be given to 'Anonymous Referee' and the source.
The images or other third party material in this Peer Review File are included in the article's Creative Commons license, unless indicated otherwise in a credit line to the material. If material is not included in the article's Creative Commons license and your intended use is not permitted by statutory regulation or exceeds the permitted use, you will need to obtain permission directly from the copyright holder.
To view a copy of this license, visit https://creativecommons.org/licenses/by/4.0/

Authors' Response Manuscript "Unified down-stream analysis of crosslinking mass spectrometry results with pyXLMS"

We want to thank the reviewers for taking the time and effort to review our manuscript and provide detailed feedback. We are very grateful for the reviewers' comments which have substantially improved the quality of our work and this manuscript.

Below follows a point-by-point response to the comments made by the reviewers. The original reviewer comments are followed by our response in blue.

Reviewers' comments:

Reviewer #1

(Remarks to the Author):

The manuscript "Unified down-stream analysis of crosslinking mass spectrometry results with pyXLMS" presents a useful and timely framework to make intuitive the analysis across different search engines. The description of the data processing is clear and informative, and the algorithm is said to speed up the post-analysis not only for bioinformaticians but also for non-bioinformaticians. However, important aspects of the post-analysis, interoperability, and implementation details need clarification or correction to match the claims made in the text and abstract.

We thank the reviewer for their efforts, the positive assessment of our work, and especially their extensive feedback which helped us a lot in improving the quality of the software and the manuscript!

1) Level of analysis: CSM/XL vs PPI
The manuscript repeatedly states that pyXLMS focuses on CSM and XL aggregation and analysis. However, current XLMS applications place strong emphasis on PPI level questions (interactomes, network perturbation, ligand or toxin targeting, etc.), and this is also how the method is motivated in the abstract and introduction.
• The current description does not clearly explain if or how CSM and XL information is systematically propagated to PPI level (e.g. how protein–protein interactions are defined, how ambiguous peptides are handled at PPI level, or how multi protein groups are treated).
• FDR control at PPI level is not described, whereas FDR is discussed only for CSM and XL levels and only for a subset of engines (MaxLynx, MS Annika, xiSearch).

In this context, I would suggest to:
a) explicitly describe how pyXLMS derives PPIs from XLs: rules for grouping crosslinked residue pairs into protein–protein interactions, how ambiguity is handled, and how intra vs inter protein links are converted into network edges.
b) clarify whether PPI level FDR is implemented, and if not, state this limitation clearly and discuss how users are expected to control error rates at PPI level (e.g. via external tools, recommended workflows, or future work).
c) If PPI output is not supported, temper statements in the abstract and introduction regarding "identification of protein–protein interactions" to make clear that pyXLMS currently operates at CSM/XL level and relies on downstream tools to infer PPIs.

We apologize that this was not made clear enough in the manuscript, pyXLMS exclusively works on CSM and XL level and does not support PPIs directly. This decision was made because we currently do not support any down-stream analysis tools that would require PPIs as input (and have not encountered such a tool yet). Some of the down-stream analysis tools might output PPIs using the inputs generated by pyXLMS, but handling aggregation and validation is then happening on the side of the down-stream analysis tool. We might support PPI input in the future if we see enough use cases but currently have no plans to implement them.

We have gone through the abstract and introduction again and PPIs are only mentioned as being applications of XLMS and not in the context of pyXLMS, we apologize if that came across wrong. We have added a statement in the pyXLMS description and in the limitations that explicitly clarifies that pyXLMS does not support PPIs directly at the moment.

Actions taken:

Manuscript: We explicitly added that pyXLMS does not directly support PPIs as a limitation in both the pyXLMS description in the introduction, results, and in the discussion.

Software: We have added a FAQ (see https://pyxlms.dev/docs/faq) that specifically clarifies which aggregation levels pyXLMS supports and that PPIs are not supported.

2) mzIdentML adoption and positioning

The introduction claims that mzIdentML 1.2/1.3 has "not seen wide adoption in the field of XLMS with only a handful of crosslink search engines and down-stream analysis tools supporting the format." At the same time, you list mzIdentML 1.2 support in pyXLMS and emphasize interoperability as a main contribution. Given that many current engines do export mzIdentML 1.2/1.3, this statement feels outdated and undersells the existing ecosystem.

In this regard please:

a) Re evaluate and soften the statement on "not seen wide adoption," or back it up with an up to date, quantitative overview of which crosslink engines and viewers support mzIdentML 1.2/1.3.

While we acknowledge that "not seen wide adoption" may be a harsh statement, please consider the tables below (Table R1 and Table R2) listing which of the crosslink search engines/tools support mzIdentML export and which down-stream analysis tools support mzIdentML import. These are not exhaustive lists by any means but rather what is included in pyXLMS. However, most of these tools are quite popular in the field. Only 4/9 tools support mzIdentML export and only 4/10 tools support mzIdentML import. Considering the total number of combinations of crosslink search engines and down-stream analysis tools, this means that only 16/90 (or ~18%) of combinations support mzIdentML. The remaining 82% would have to be done via custom result transformations if pyXLMS did not exist. We also want to note here that it is not always clear what exactly is exported to mzIdentML by the crosslink search engines, e.g. some crosslink search engines export raw results (e.g. Proteome Discoverer/MS Annika reports raw results but target-target matches only) while others only export validated results (e.g. Scout). Because mzIdentML is not human readable it is also hard to quickly verify this (or also if other filtering methods have been applied or not). Additionally, support for mzIdentML version 1.3 is still a work-in-progress in many cases, e.g. xiVIEW which is arguably one of the most used tools in the field currently does not yet support mzIdentML version 1.3, Proteome Discoverer also does not yet support the newest version as far as we are aware which affects MS Annika (developed by our lab) and XlinkX!

Nevertheless, we have slightly softened the statement in the manuscript for fairness.

Actions taken:

Manuscript: We have slightly softened the statement on mzIdentML adoption.

Crosslink Search Engine/Tool	Exports mzIdentML?
**MaxQuant/MaxLynx**	No
**MeroX**	No
**MS Annika**	Yes (mzIdentML v1.2)
**pLink**	No
**Scout**	Yes
**xiSearch**	No
**xiFDR**	Yes
**xiNET/xiVIEW**	No
**XlinkX**	Yes (mzIdentML v1.2)

Table R1: *Overview of mzIdentML adoption for tools exporting crosslink results.*

Down-stream Analysis Tool	Imports mzIdentML?
**AlphaLink2**	Yes
**IMP-X-FDR**	No
**ProXL**	No
**PyXlinkViewer**	No
**xiFDR**	Yes
**xiNET**	No
**xiVIEW**	Yes (mzIdentML v1.2)
**XlinkDB**	No
**xlms-tools**	No
**XMAS**	Yes

Table R2: *Overview of mzIdentML adoption for tools importing crosslink results.*

b) Highlight that pyXLMS can act as a bridge layer on top of the emerging mzIdentML 1.3 crosslink support, rather than implying the standard is rarely used.

Please see the answer to the point below.

c) Consider recommending mzIdentML 1.2/1.3 as a default interchange format for XLMS, with pyXLMS adding convenience and export functions on top of it.

We thank the reviewer for their suggestion; we have clarified that pyXLMS is not a replacement for mzIdentML and that mzIdentML should be used whenever possible. We have emphasized that pyXLMS should be seen as an extension for cases where usage of mzIdentML is not possible or not preferrable. Ultimately, our goal is simply to empower researchers in all situations – whether mzIdentML is available or not.

Actions taken:

Manuscript: We have added clarification to the manuscript.

Software: We have added clarification to the software, specifically in the FAQ section (https://pyxlms.dev/docs/faq) which now explicitly contains "Is pyXLMS an alternative to mzIdentML" to position pyXLMS better.

3) FDR and engine coverage

The manuscript states that FDR estimation in pyXLMS is “currently only the case for MaxLynx, MS Annika, and xiSearch,” because these provide targets/decoys and a single score. However, Scout is also described as providing CSV output and is included among the supported engines, and you mention reading both CSM and XL levels for Scout.

In the current XLMS landscape, Scout can produce target/decoy information at CSM, XL, and PPI levels, and it would be important to clarify if and why this is (not) used in pyXLMS. If technical reasons prevent using Scout’s outputs for FDR, explain the limitation explicitly (e.g. missing combined score, multiple scoring dimensions, or format specifics). In addition, discuss whether PPI level FDR is planned, in particular leveraging engines like Scout that provide decoy PPIs.

We thank the reviewer for the additional input on Scout. As far as we understood Scout does not use a single score for FDR estimation (e.g. “missing a combined score”) but rather combines several scores for validation. This is mentioned by the developers on GitHub in this issue (screenshot included below):

https://github.com/diogobor/Scout/issues/15#issuecomment-3293380290

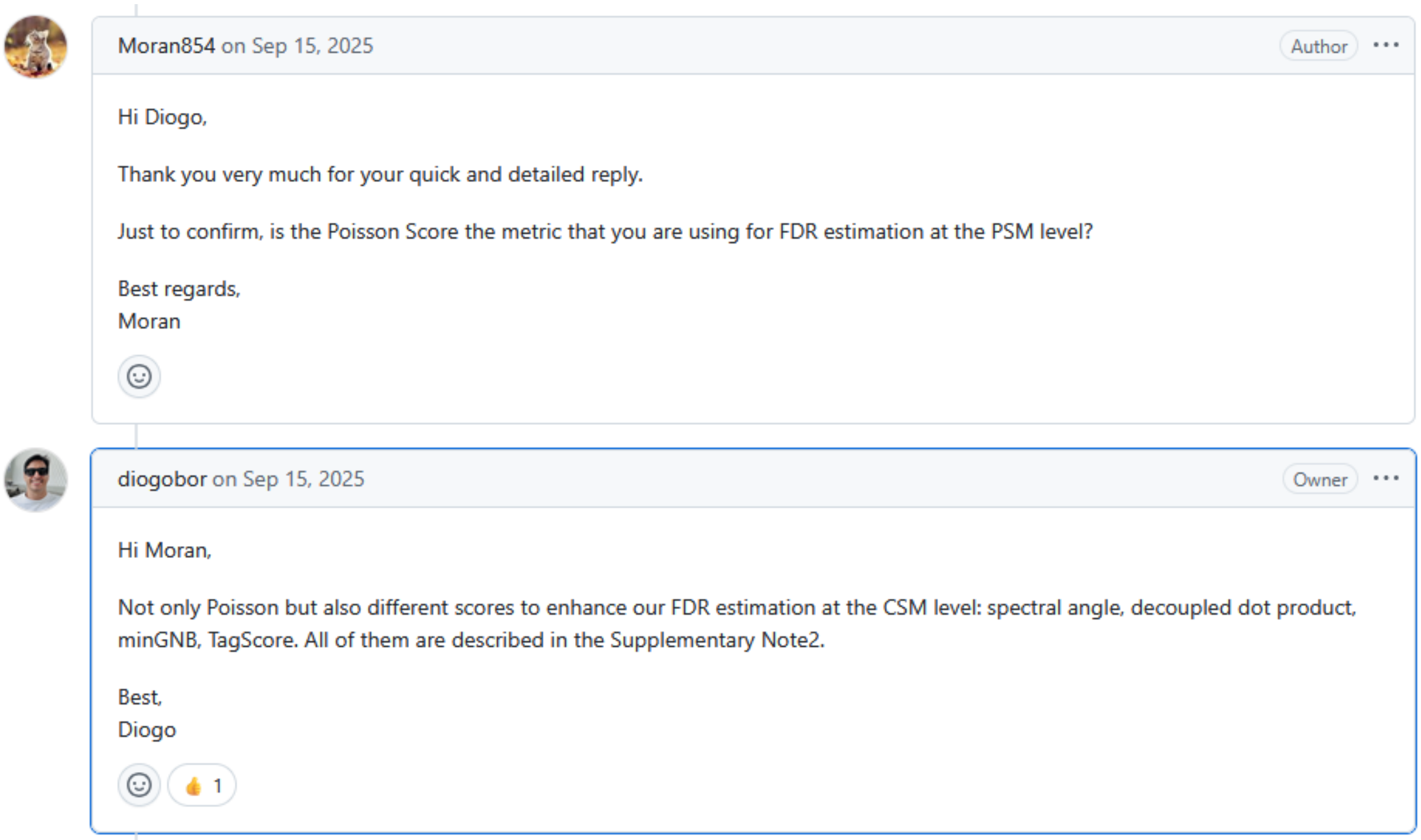
Moran854 on Sep 15, 2025 Author

Hi Diogo,

Thank you very much for your quick and detailed reply.

Just to confirm, is the Poisson Score the metric that you are using for FDR estimation at the PSM level?

Best regards,
Moran

diogobor on Sep 15, 2025 Owner

Hi Moran,

Not only Poisson but also different scores to enhance our FDR estimation at the CSM level: spectral angle, decoupled dot product, minGNB, TagScore. All of them are described in the Supplementary Note2.

Best,
Diogo

We are happy to correct this in pyXLMS/the manuscript if the reviewer has more information!

Additionally, we want to highlight that validation of Scout results is technically possible with pyXLMS, pyXLMS reads the “XLScore” from the unfiltered results and the target-decoy information. However, since the validation in pyXLMS depends on a single score (in this case the “XLScore”) the results will be different than the validation by Scout itself. We therefore did not include Scout in the list of supported search engines for validation in pyXLMS.

Regarding PPIs please refer to the answer given in 1).

Actions taken:

Manuscript: We have clarified why Scout is excluded from the list of recommended search engines for pyXLMS validation.

4) Intersection of multiple engines in the web app
The manuscript claims that pyXLMS "allows to easily create intersections of CSMs and XLs, either from different replicates or different crosslink search engines." In the web application description, users are guided through upload, filtering, visualization, and export.
In practice, when using the public web app, the upload step enforces selection of a single engine/format from a dropdown, making it unclear how intersections across different search engines can be created in the GUI workflow you advertise for non programmers.

In this context, I would suggest clarifying whether multi engine intersections are currently only available in the Python API, and if so, state this explicitly and adjust the claims about the web app accordingly. Additionally, provide a brief and explicit example (user interface steps or code) demonstrating how to obtain the intersections of XLs in pLink, Scout, and XlinkX for the Cas9 dataset.

We thank the reviewer for pointing this out! Indeed, intersections are only possible via the python package and not in the web app. We have added a clarification to the manuscript and added a section to the Supplementary Information titled "Web App Feature Support" that explicitly lists all the functions that are not available in the web application. Additionally, we have listed where users can find step-by-step python instructions for the features that are not available in the web app, including the complete code that is used for this manuscript to recreate the intersection and reproduce all the results.

Actions taken:

Manuscript: We have added clarification to the manuscript and added a new section to the Supplementary Material that describes which features are exclusively available in the python package.

Software: The documentation now also explicitly lists which features are not available in the web application, e.g. refer to the user guide → Documentation → Web Application → Feature Support , or this link:

https://pyxlms.dev/docs/webapp/features.

5) Export options vs Table 2 (xiView, MS Annika)
Table 2 lists xiVIEW as a supported export format (XL only) and does not mention MS Annika as an export target. The text emphasizes "more than ten different down-stream analysis tools and formats" and cites xiVIEW as a primary example. In the public web app, however, the export options presented to the user include only IPM X FDR, MS Annika, ProXL and xiFDR, with xiVIEW not available and MS Annika not listed in Table 2.

So, I would suggest reconciling Table 2, the main text, and the web app implementation. Ensure that xiVIEW export is consistently available in the web app if claimed in the manuscript or explicitly mark it as "currently only via Python API" if that is the case. In addition, add a note in the Methods or Implementation section stating that the set of export formats in the hosted web app may lag behind the Python package and specify what is guaranteed at the time of publication.

We thank the reviewer for bringing this to our attention. We failed to clarify in the manuscript that the export options largely depend on what data is uploaded, e.g. the export options IPM X FDR, MS Annika, ProXL and xiFDR were visible because CSMs were uploaded. xiVIEW was not listed as an

export option because it needed crosslinks (so CSMs aggregated to peptide pairs or residue pairs) as input – this is why xiVIEW was also listed as XL only in the Table 2. We have now clarified this in the manuscript and now additionally also support CSMs to be exported to xiVIEW. We also point out in the manuscript now that export options that require crosslinks can be used with CSMs by selecting "aggregate crosslink spectrum matches to crosslinks" in the "Load Data" tab in the web application. We also added this to the FAQ because it might be a common question for users.

Regarding why MS Annika is not mentioned as an export target: MS Annika is not a down-stream analysis tool, we simply added it as convenience because we have developed tools in the past that require the MS Annika format. This option will only be useful in very niche cases and therefore decided not to highlight it.

Actions taken:

Manuscript: We have added clarification regarding the availability of export options.

Software: pyXLMS now also supports CSMs for export to xiVIEW, additionally we have clarified the behavior of export options in the FAQ (https://pyxlms.dev/docs/faq).

6) Versioning and Scout choice for the benchmark
Section 4.1 states that you tested Scout version 1.6.3 (Borges repository) and Scout 2.0.0 beta (Liu Lab repository). In the data analysis section, you report using Scout 2.0.0 beta from the Liu Lab for the Cas9 benchmark. For many users, the stable Scout 1.6.3 (or 2.0.0) from the Borges repository is now a practical default and, in some datasets, performs better than the beta variant.
In this case, justify the exclusive use of the beta 2.0.0 version in the main analysis; at minimum, mention that alternative releases (e.g. Scout 2.0.0 from Borges) exist and might produce different or improved results.
Moreover, if feasible, re run the Cas9 dataset with the stable Scout 2.0.0 (Borges) and report CSM, XL, and PPI numbers at 1% FDR alongside the existing pLink and XlinkX results. If re analysis is not possible, explicitly discuss how sensitive your conclusions (e.g. relative performance of pLink vs Scout) might be to the specific Scout version used.

We apologize if we did not use the correct Scout version, we simply used the newest version available at the time, which was at that point the Scout 2.0.0 beta version from the Liu lab. We have now re-run the searches with the newest version from the Borges repository (version 2.1.0), please find a comparison shown in the figures below (Figure R1 – R3).

The raw Scout results from the re-run can be found here:

https://github.com/hgb-bin-proteomics/pyXLMS-manuscript/tree/master/results/scout-re

The complete code for comparison of the two Scout versions can be found here:

https://github.com/hgb-bin-proteomics/pyXLMS-manuscript/blob/master/02_scout_comparison.ipynb

Moreover, we made sure that the numbers read with pyXLMS are consistent with the numbers in the exported CSV files to rule out any kind of parsing errors in pyXLMS.

We have concluded that the Liu lab version performs better in terms of number of identifications for this dataset. As to why there is this large difference between the two versions we have no clear answer, but from what we understood is that there are slight differences in FDR estimation which might affect the results.

In any case we want to emphasize – and we also mention this in the manuscript now – that what we report should not be taken as a benchmark of the different crosslink search engines, e.g. we do not investigate the results for false positives and correct FDR estimation. Our goal is simply to show how pyXLMS can easily give an overview of the results for all these search engines and combine their results.

Actions taken:

Manuscript: We emphasize in the manuscript that these results are not supposed to be benchmarks and that different Scout versions exist. We have not changed the numbers for Scout as we think that it is beneficial for Scout to use the higher (arguably better) numbers from the original search.

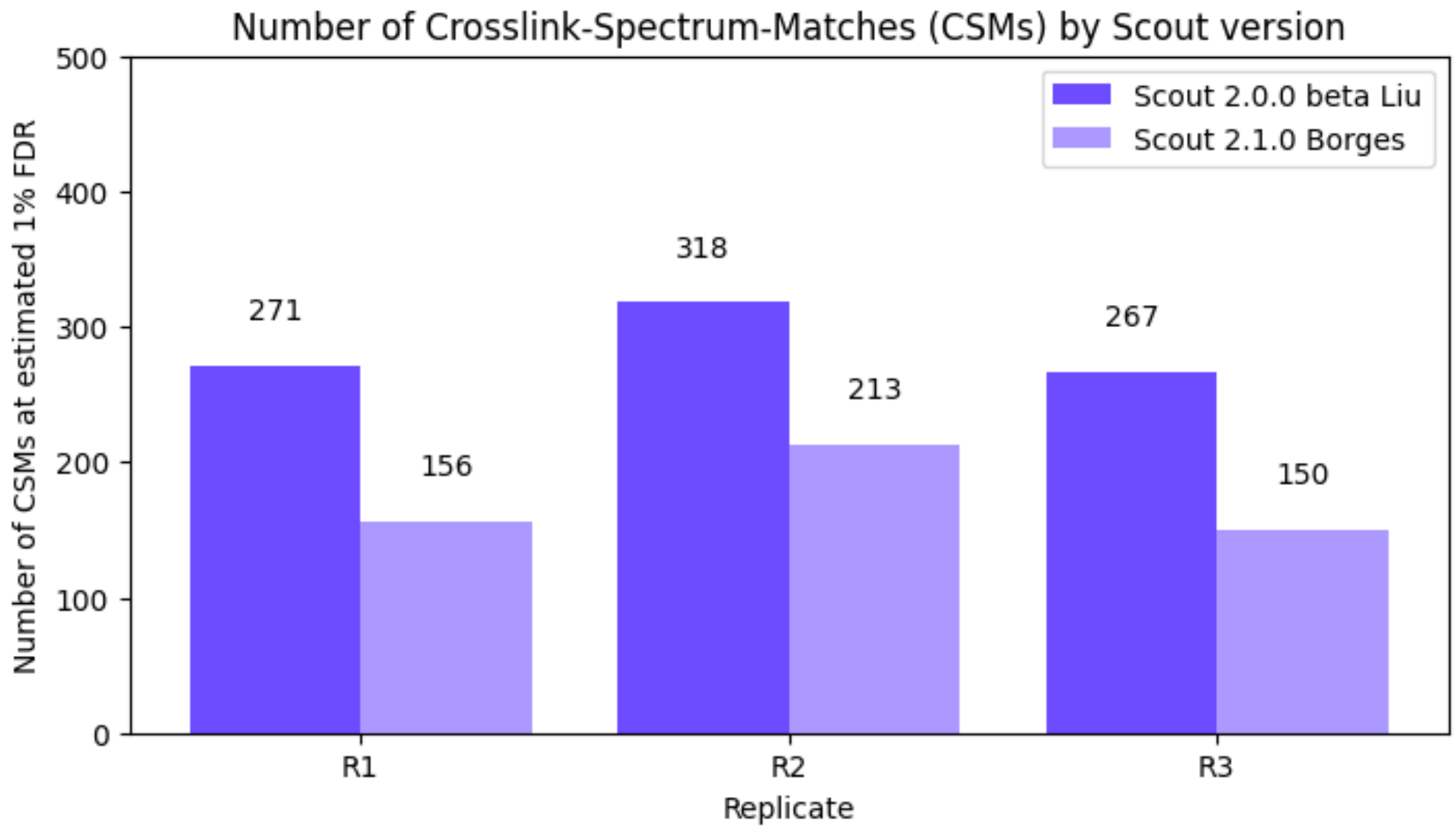

Figure R1: *Comparison of the two Scout versions in terms of number of CSMs at 1% estimated CSM-level FDR for each of the three replicates.*

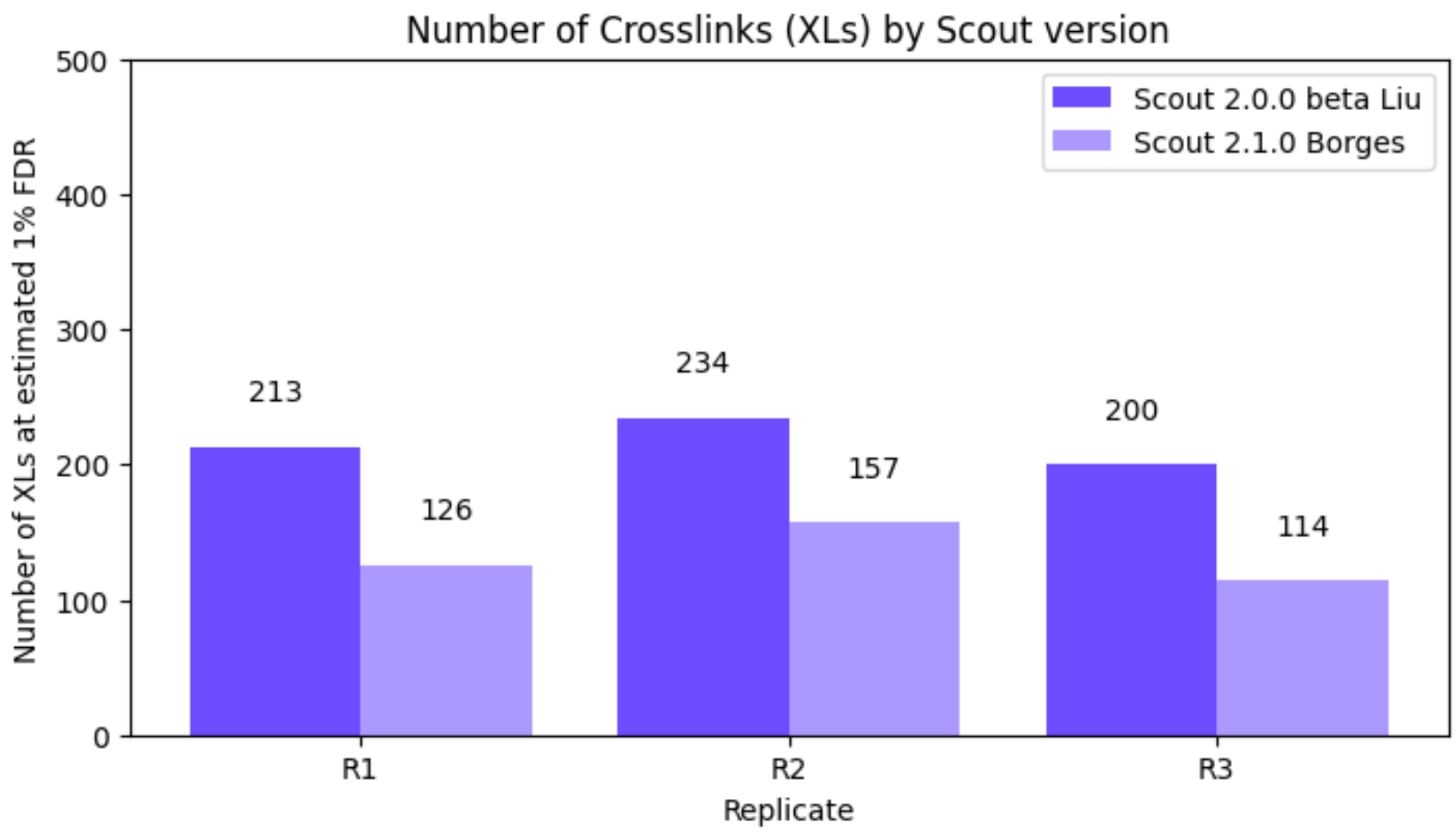

Figure R2: *Comparison of the two Scout versions in terms of number of unique residue pairs at 1% estimated residue pair-level FDR for each of the three replicates.*

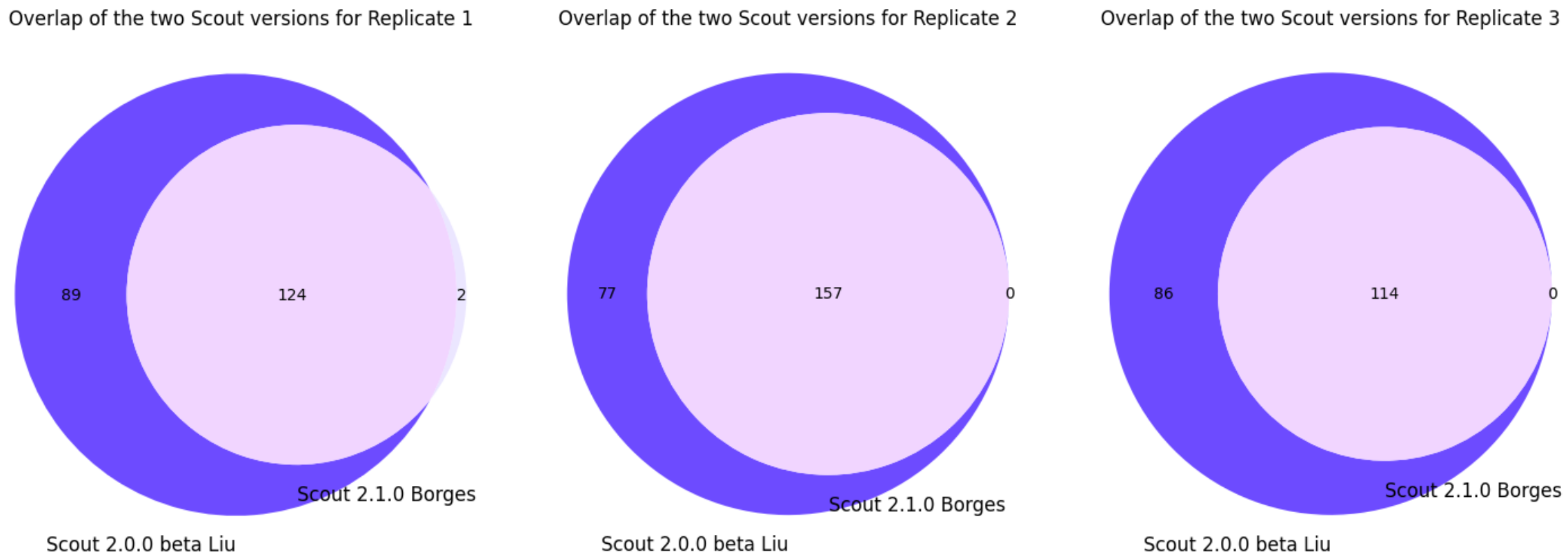

Figure R3: *Overlaps in residue pairs between the different Scout versions for each replicate. The Scout version from the Liu Lab identifies all residue pairs of the Scout version from Borges (with the exception of replicate 1, where 2 residue pairs are exclusively identified by the Borges version). The Liu Lab version also identifies an extra 77 to 89 residue pairs on top. (All results at 1% estimated residue pair-level FDR).*

7) Installation requirements and reproducibility

The Implementation section details Python packages and testing (pandas, pyteomics, biopython, matplotlib, biopandas, lxml, streamlit, sphinx, etc.) and reports 584 tests covering parsing and core functionality. However, minimal system requirements are not stated explicitly. However, there are a lack information about the package versions used during the development as well as the minimum set up for the machine. In this context, add a short subsection (e.g. “System requirements”) specifying at least: minimum Python version (e.g. 3.9+), required operating systems (Linux, macOS, Windows) and tested versions, approximate RAM/disk requirements for typical XLMS datasets, and any known performance constraints.

We apologize that this information was missing from the manuscript and was only described in the online documentation where it was not visible enough/easy enough to find. pyXLMS (including the web application) runs on any operating system as it is python native, it supports any python version from 3.7 or greater, however we do recommend a python version that is in the active support cycle and considering the performance improvements of python 3.14, an up-to-date installation makes sense especially for large datasets. There are no specific requirements in terms of hardware or any performance constraints, most functions in pyXLMS should complete instantly even for large datasets. File reading times depend on the file format and size of the file but usually only take longer once files reach several GB in size, which is usually not the case for crosslinking results. mzIdentML file parsing may take a long time for results containing > 10 000 identifications, this is mostly because XML parsing is rather slow in python. Validation and re-annotation of crosslink positions may also take a few minutes for larger (> 100 000 CSMs/XLs) datasets. We generally recommend using the python package instead of the web application for large datasets because the python package shows progress bars for every step and therefore allows accurate tracking, this cannot be seen in the web application interface.

We did not use fixed package versions during the development of pyXLMS, this is also not possible when targeting all python versions starting from version 3.7 – instead our CI pipeline tested pyXLMS with the most recent package versions for each python version automatically.

We use fixed package versions for the web application which can be found here (we use uv [https://docs.astral.sh/uv/] for project and dependency management):

https://github.com/hgb-bin-proteomics/pyXLMS/tree/master/gui

We have also used fixed package versions for all analyses of this manuscript, they can be found here:

https://github.com/hgb-bin-proteomics/pyXLMS-manuscript

For users who want to work with pyXLMS we now also offer a template python project with an already setup environment and fixed package versions:

https://github.com/hgb-bin-proteomics/pyXLMS-template

Actions taken:

Manuscript: We have added minimum requirements to the manuscript and referenced where users can find more information.

Software: We have created a template pyXLMS project with fixed package versions that users can directly build upon. We have added some recommendations in terms of performance, e.g. when to use the python package instead of the web application to the documentation.

On the other side, for the web app, clarify what is needed to run locally or self host (Python version, streamlit version, browser compatibility). Since the authors emphasized non expert usability, make sure the user guide (cited URLs) includes a minimal "one page" install recipe that matches the versions used in this manuscript.

We have a one page install recipe in the user guide via Documentation → Web Application → Introduction, or directly via the link https://pyxlms.dev/docs/webapp

Minor comments and suggestions
1. Consistency in engine and format lists
The text at multiple points counts "seven different crosslink search engines" but then lists MaxLynx, MeroX, MS Annika, pLink 2/3, Scout, xiSearch/xiFDR, and XlinkX, which reads as more than seven logical tools depending on how they are grouped. For clarity, define whether xiFDR and XlinkX are treated as separate input engines or as auxiliary formats.

We have changed wording to "more than seven" and "several" throughout the manuscript since the number has already changed and will change frequently anyway. We originally grouped pLink 2 and 3, as well as xiSearch and xiFDR together.

Actions taken:

Manuscript: Changed the wording to "more than seven" and "several" instead of a fixed number.

2. Clarify the mzIdentML version support
The authors explicitly mention support for mzIdentML 1.2 in the tests, and cite mzIdentML 1.3 as a format that extends crosslink reporting. It would be helpful to state clearly whether 1.3 is fully supported as input (and tested) or treated as 1.2 with partial coverage.

Actions taken:

Manuscript: We added that we also tested mzIdentML 1.3.

Software: We have added full support for mzIdentML 1.3 and have now extensively tested the mzIdentML parser using all official HUPO PSI examples for v1.2 and v1.3, as well as mzIdentML files from MS Annika, XlinkX, and Scout (both v1.2 and v1.3).

3. Documenting limitations more prominently
The Discussion already notes that lack of decoys in some engines prevents validation and export to xiFDR and that varying information content across engines limits some exports. It would help readers if the web app and docs expose these limitations explicitly (e.g. when a user uploads MeroX results and tries to export to xiFDR, a clear message that FDR cannot be recomputed in pyXLMS).

We thank the reviewer for their suggestion!

Actions taken:

Software: Limitations have been added directly to the README file in the repository and to the front page of the documentation. We also added an information/warning banner to the web application when the user selects validation with any of the non-supported crosslink search engines.

4. Terminology
Ensure consistent use of terms like "crosslink/residue pair," "XL," and "PPI" throughout the text and figures and clarify early on that the core data structures operate at CSM and XL levels, with PPIs derived rather than directly stored.

Actions taken:

Manuscript: We defined terms as early as possible in the manuscript and made sure their usage is consistent. We added clarification on the used data structures in pyXLMS and mention that pyXLMS does not directly support PPIs.

(Remarks on code availability):

I would suggest to create unit_tests for the code. For instance, src/pyXLMS/exporter requires unit tests for each parser.

We already have unit tests for all parsers and all exporters, our phrasing in the manuscript that states "generally, the export to all down-stream analysis tools should work for all crosslink search engines and input formats…" with the emphasis on "should" might be a bit misleading as we do not describe a potential technical mishap here. Export would simply fail if an input file does not contain some of the information that is required for a specific export, e.g. consider an export that needs residue pair information but the input file does only contain peptide sequences with peptide crosslink positions (without proteins and protein crosslink positions). The export would fail because that information is not available, however failing there is by design as there is no way to recover without alerting the user and taking extra steps (e.g. reannotation with pyXLMS).

Another suggestion would be to call pre commit hook before committing the code to make the repository more clear.

We thank the reviewer for their suggestion! We prefer GitHub Actions over pre commit hooks which are triggered on pull requests. The CI pipeline takes considerable amount of time and would be overkill as a pre commit hook.

Reviewer #2

(Remarks to the Author):

In this manuscript, the authors present their pyXLMS tool that could essentially re-format output from different crosslink search engines, compatible with different downstream analysis tools. This tool is helpful especially for experimental researchers by reformatting their XLMS data so that they can input that data to other published analysis tools. Though the authors claim that their tool can also perform data analysis, the features that constitute this claim are mainly for data summarization rather than significant data analytical capability. As described in detail in the comments below, this tool is of limited use for the field and not suitable for publication in Nature Communications:

We thank the reviewer for their time and effort and their valuable feedback on our work. We acknowledge that the term "data analysis" in itself might not have been the most appropriate to describe pyXLMS, however we would argue that data summarization and post processing like filtering, grouping, and annotation, as well as quality control such as score distributions and validation are equally valuable and needed methods for further down-stream analysis and hence of utmost use for eventual data interpretation. We have extended upon these points with suggestions by the reviewer, including more quality control metrics such as crosslink-spectrum-matches per peptide pair or per residue pair, and integration with STRING to investigate how well results match with known interactions.

Considering that we already had positive interactions (some of them even publicly trackable on GitHub, e.g. see https://github.com/hgb-bin-proteomics/pyXLMS/issues/201 and https://github.com/hgb-bin-proteomics/pyXLMS/issues/240) with several researchers in the field who were not involved in the project, even though pyXLMS has not been officially published yet, we would also argue that pyXLMS does have wide applicability as it serves as the missing link across a multitude of data analysis and down-stream tools used in the community. This makes pyXLMS especially valuable for non-bioinformaticians as well as scientists new to the XLMS field. Especially taking into account that there is currently no other software doing something similar.

Actions taken:

Manuscript: We have rephrased some of the manuscript to position pyXLMS better, e.g. not as a standalone down-stream analysis tool but rather as an important middle layer that is needed to bridge the gap between crosslink search engines and down-stream analysis tools.

(a) The manuscript initially focuses on how their tool allows data reformatting and summarization on an example dataset. Later, the authors input this reformatted data to multiple published tools such as xiVIEW, AlphaFold2, AlphaLink2 and report and compare the results. The entire Figure 3 is dedicated towards showing these results and the corresponding text sounds like a tutorial for using these other tools. Additionally, Figure 2 and 4 essentially show the visualization of the data summary, which could just be presented as a single figure. Finally, Figure 5 just shows a snapshot of the tool's user guide, which does not qualify as a full figure.

The result we want to portray is that with pyXLMS an analysis like that is now possible with a few lines of code instead of writing several separate scripts for each crosslink search engine and down-stream analysis tool. This workflow also included grouping, re-annotation of protein crosslink positions, and intersections of results, which all fitted into one relatively small jupyter

notebook with ~70 cells (which also still contains a lot of redundant statements which are simply there for illustration purposes). The notebook can be found here:

https://github.com/hgb-bin-proteomics/pyXLMS-manuscript/blob/master/01_figure_code.ipynb

However, showcasing this result in a manuscript is inherently challenging without also showing the final down-stream analysis results (as given in Figure 3). We apologize if that comes off a bit like a tutorial, although we think that is not necessarily a bad thing. Especially since our software is also aimed at newcomers. Figures 2 and 4 have been directly created with pyXLMS and Figure 4 is separate from Figure 2 because it also showcases the web application, which is a major selling point of our software. We recognize that Figure 5 might not qualify as a full figure and have moved it to the Supplementary!

Actions taken:

Manuscript: We have moved Figure 5 to the Supplementary.

(b) I tested the webserver (http://pyxlms.projekte.fh-hagenberg.at) using an example dataset from their repository (https://github.com/hgb-bin-proteomics/pyXLMS/blob/master/data/ms_annika/XLpeplib_Beveridge_QEx-HFX_DSS_R1_CSMs.txt). When I clicked run, it started running but threw the following error:
"Something went wrong! This is most likely due to missing information in the results!
KeyError: 'st.session_state has no key "aggregated". Did you forget to initialize it? More info: https://docs.streamlit.io/develop/concepts/architecture/session-state#initialization'
Traceback:
File "/pyXLMS/gui/streamlit_app.py", line 642, in input_tab
if st.session_state["aggregated"] is not None:
~~~~~~~~~~~~~~~~^^^^^^^^^^^^^^
File "/usr/local/lib/python3.12/site-packages/streamlit/runtime/state/session_state_proxy.py", line 101, in __getitem__
return get_session_state()[key]
~~~~~~~~~~~~~~~~~~~^^^^^
File "/usr/local/lib/python3.12/site-packages/streamlit/runtime/state/safe_session_state.py", line 104, in __getitem__
return self._state[key]
~~~~~~~~~~~^^^^^
File "/usr/local/lib/python3.12/site-packages/streamlit/runtime/state/session_state.py", line 478, in __getitem__
raise KeyError(_missing_key_error_message(key))"
Looking at the error message, I guessed perhaps it was because I selected "Validate aggregated crosslinks via FDR estimation option" but did not select "Aggregate crosslink spectrum matches to crosslinks" option. Once I selected it too, the error was gone. The server needs to be thoroughly tested for such inconsistencies especially because it was aimed at helping the experimental researchers and we cannot expect them to trouble-shoot based on the trace-back error log.

We thank the reviewer for finding and reporting this bug! It is fixed in the newest version of the pyXLMS web application. We are sorry that it slipped through our testing. We had 5 people from 3 different research groups working on extensively testing the web application, however it is unfortunately always possible that bugs are not caught during testing. Even considering that the pyXLMS codebase currently has 85% test coverage with more than 650 test cases, there might be
~~~~~~~~~~~

cases that simply nobody has thought of yet. We do want to emphasize though that we are happy to receive bug reports, questions or feedback at any time, either via GitHub issues, discussions, or via personal email and are usually quick to respond!

Actions taken:

Software: The corresponding bug has been fixed as of pyXLMS-GUI version 1.4.1!

(c) Question about the definition of interprotein crosslinks: For the example I tested, there was an "intraprotein" CSM with both peptides alpha and beta as "GQKNSR" and the position of that crosslink in protein is also identical (779), indicating that the crosslink occurred between two different molecules of Cas9 protein. In this case, shouldn't it be classified as "interprotein", since it is between to different protein molecules albeit the same id? Example for a different but similar case is crosslinks between different subunits of a homo-oligomeric protein.

The reviewer is right that these are special cases which are sometimes referred to as "homomeric interlinks" depending on the search engine and/or researcher. For example, the crosslink search engine pLink would label a homomeric interlink as an intralink, the crosslink search engine Scout specifically labels them as homomeric interlinks. (And we do not know of any example that would label them as "normal" interlinks.) pyXLMS labels them as intralinks because for analysis they are usually grouped together with intralinks and pyXLMS calculates link type simply by checking if the associated proteins of both crosslinked peptides overlap (intralink) or not (interlink). It should also be noted that technically any intralink of a homo-oligomeric protein could be an interlink, there is simply no way to tell without also looking at the structure (if it exists). Homomeric interlinks are just the special cases where we are sure just from the sequence alone that an intralink is actually a (homomeric) interlink.

Actions taken:

Manuscript: We have clarified that pyXLMS labels homomeric interlinks as Intralinks.

(d) It would be helpful to the users if the crosslink level table has an additional column showing how many CSMs were aggregated to give that crosslink.

We thank the reviewer for the suggestion! Because this is specifically only possible if crosslinks are aggregated within pyXLMS itself we cannot make it a general solution. However, we have now implemented two visualizations in the web application that do something similar. Additionally, we have added two filtering options in the python package that correspond to these visualizations. Specifically, plotting the number of CSMs per peptide pair, and the number of CSMs per residue pair, as shown in the Figure below (Supplementary Figure 1) which has also been added to the Supplementary Information. The figure shows how this now looks in the web application. The pyXLMS python package gives more nuanced control and allows filtering by peptide pair or residue pair, which would make it possible to – for example – only aggregate CSMs to peptide pairs where peptide pairs have at least X number of supporting CSMs. Filtering in the python package is further described with step-by-step instructions here: https://pyxlms.dev/docs/transform/filter

Actions taken:

Manuscript: We have added the new filtering and visualization options to the manuscript and added a corresponding supplementary figure to the supplementary material.

Software: We added new filtering and visualization functions that allow deeper insights into the number of CSMs per peptide pair or residue pair.

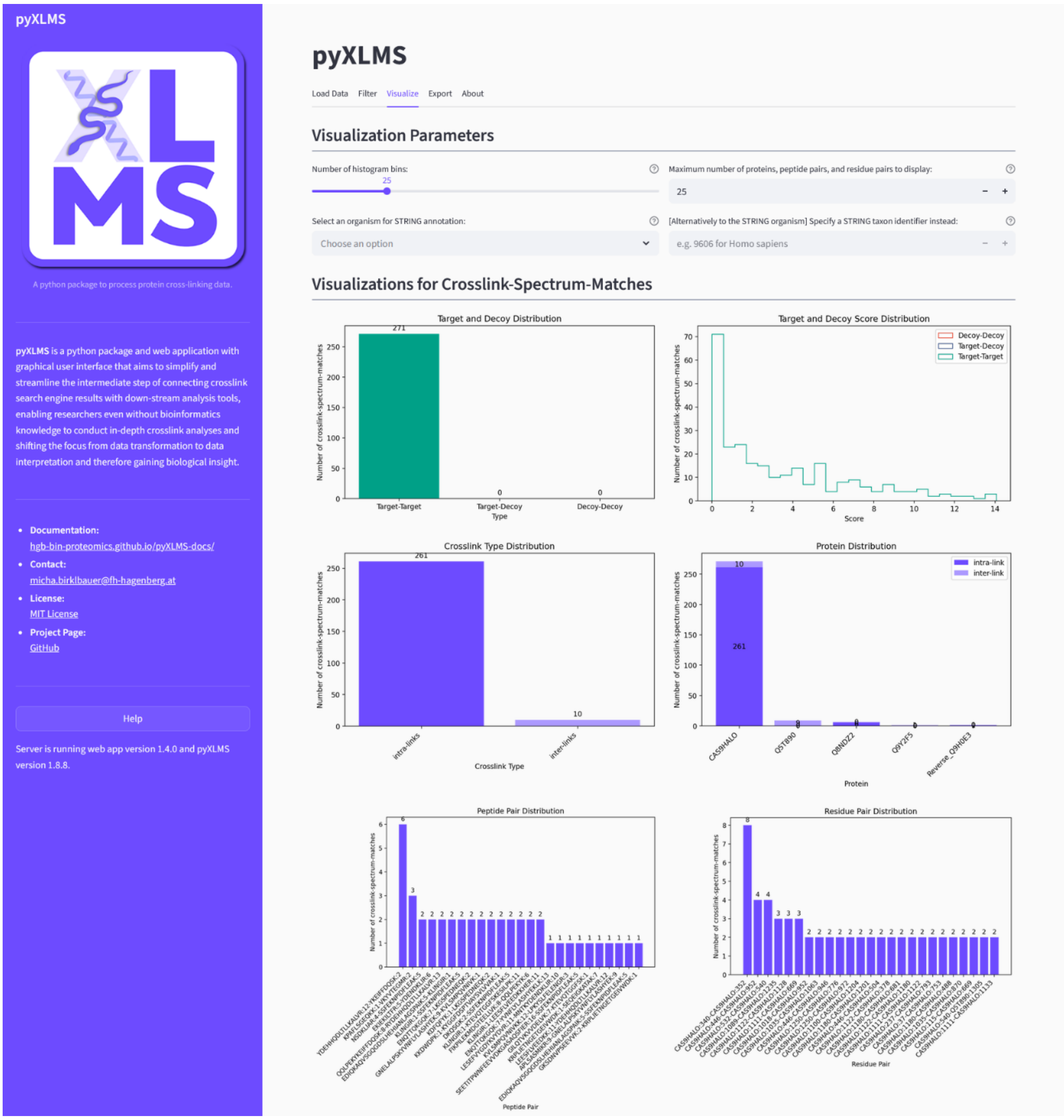

Supplementary Figure 1: *Overview of the visualization tab in the pyXLMS web application for crosslink-spectrum-matches (CSMs). Users can control the visualization, e.g. the number of histogram bins and the number of shown proteins/peptide pairs/residue pairs via the parameters at the top. The first panel shows an overview of the target-target, target-decoy, and decoy-decoy matches which in this case only shows a bar for target-target CSMs because the results were already validated and decoy free. The second panel shows the target-decoy score distribution. The third panel shows the number of intra- and inter-link CSMs, while the fourth panel shows the number of intra- and inter-link CSMs per protein. The fifth panel shows the number of CSMs per unique peptide pair, while the number of CSMs per unique residue pair is shown in panel six. Visualizations are shown for validated results of Scout using replicate one of the Cas9 dataset.*

This figure can be reproduced by uploading the file (Scout, DSBSO) to the web app:

https://github.com/hgb-bin-proteomics/pyXLMS-manuscript/blob/master/results/scout/Cas9_HeLa_Cyt_r1/Cas9_HeLa_Cyt_r1_filtered_CSMs.csv

Additionally, the "Completeness" column in the CSM table has "partial" for all entries and the crosslink table has "complete" for all the entries. Could the authors explain the difference? I tried to find an explanation for this column in the tutorial but was unable to find it.

This is simply a convenience field for programmatic access, completeness equal to "full" would mean that the CSM/XL does not have any "None" values in any of the fields, therefore containing "full" information. This is mostly to avoid excessive checking for "None" values whenever a specific function requires specific fields. As an example, the export to xiVIEW requires that a crosslink has associated proteins and protein crosslink positions. If the crosslink has completeness equal to "full" the exporter will not check if the crosslink has associated proteins and protein crosslink positions. If the crosslink has completeness "partial" the exporter does an extra step (or rather four extra steps) and checks if the crosslink does have associated proteins and protein crosslink positions and will raise an exception if not. (Four extra steps because it needs to check that proteins for the alpha peptide are not None, proteins for the beta peptide are not None, protein crosslink positions for the alpha peptide are not None, protein crosslink positions for the beta peptide are not None.)

The completeness field is described in the user guide under Documentation ⇥ Working with pyXLMS ⇥ Data Types in pyXLMS, or directly at this link:

https://pyxlms.dev/docs/basics/data_types

Actions taken:

Software: We have updated the documentation and the CrosslinkSpectrumMatch and Crosslink data structures now also explicitly list the "completeness" attribute, additionally to the data types document.

(e) Page 5:"Moreover, pyXLMS is able to deduplicate and filter CSMs and XLs to only contain unique matches, something that is desired for almost all down-stream analyis methods". Is it mandatory to remove redundancy for most of the downstream analysis methods? Can the authors provide examples of such methods?

While it might not be harmful in some cases it is generally good practice to deduplicate CSMs and XLs. Consider the following examples:

We consider a CSM to be unique if its spectrum file and scan number are unique. Having non-unique CSMs would encapsule having more than one possible crosslink per spectrum, which could only be true if the spectrum is chimeric (e.g. having several different crosslinked entities in the same spectrum). This is highly unlikely. If one would now do validation, it would be possible that several of these non-unique CSMs pass the FDR cutoff, even though we know that most likely only one of any non-unique CSMs can be a true positive. In this case non-uniqueness is harmful to the results.

On the other hand, if we consider non-unique residue pairs and then plot them on a protein structure e.g. using PyXlinkViewer and pyMOL, this would then draw the same crosslink several times for each non-unique residue pair. While technically not harmful it is still not good practice (in the worst case it could create too many objects in pyMOL which might slow down the program).

Moreover, for reporting identification numbers in e.g. manuscripts, it is always required to report unique CSMs, XLs (peptide pairs or residue pairs), or protein interactions. Reporting non-unique numbers would simply inflate results and make comparison between different experimental or data analysis approaches impossible.

Finally, this increasingly becomes more important when combining results either from different replicates or even different datasets altogether where it is certain that identifications are no longer unique.

Actions taken:

Manuscript: We have added clarification to that statement in the manuscript.

(f) The authors list 'validation' of the data as one of the key functionalities of their tool but it mainly performs FDR estimation, and it is only applicable to data from a limited number of search engines as acknowledged by them (page 5: "However, validation is limited to results from crosslink search engines that report both target and decoy matches, as well as a single score for FDR estimation. This is currently only the case for MaxLynx, MS Annika, and xiSearch."). Perhaps, implementing quality assessment metrics such as the fraction of interprotein crosslinks as an indicator of data quality (PMID: 31267744) and the overlap of interprotein crosslinks with known interactions from public repositories (PMIDs: 32994567, 37071682, 34349018) could add value towards assessing the data quality.

We once more thank the reviewer for their suggestions!

The fraction of interprotein crosslinks is already implicitly available via the crosslink type visualization (and filter) as also shown in Figure 4 and the new Supplementary Figure 1 (see answer above to point d). The approach proposed by the referenced publication is unfortunately beyond the scope of pyXLMS as it requires several 10 000s of PDB structures to calculate all theoretically possible interlinks – which in itself is also computationally expensive. The approach also only specifically applies to studies involving maximally bound proteins crosslinked in crowded cellular environments (as per the authors themselves). However, comparison of intra- vs. inter-links alone is in many cases already an indicator of quality – especially in cases as presented in this manuscript where inter-protein links should basically not exist because only one protein was crosslinked.

As suggested, we have also added new functionality to pyXLMS to compare interprotein crosslinks (and CSMs) against known interactions. We decided to use STRING [1] for this purpose. We have implemented two new core functions: Firstly, one is to automatically annotate interprotein CSMs and XLs with STRING scores that can then be programmatically accessed (this includes the main STRING score as well as all sub-scores). Secondly, we implemented direct visualization of the main STRING score for interprotein CSMs and XLs in two different ways as shown in the newly added Supplementary Figure 2 (also attached below). Using this functionality requires the specification of a valid STRING organism or taxon id in the web application/python package.

Actions taken:

Manuscript: We have added how pyXLMS can be used for quality control aside of FDR validation and mention the newly added STRING integration.

Software: We implemented STRING integration that allows annotation of interprotein CSMs and XLs with all STRING scores. We also implemented two visualization options for the STRING main score.

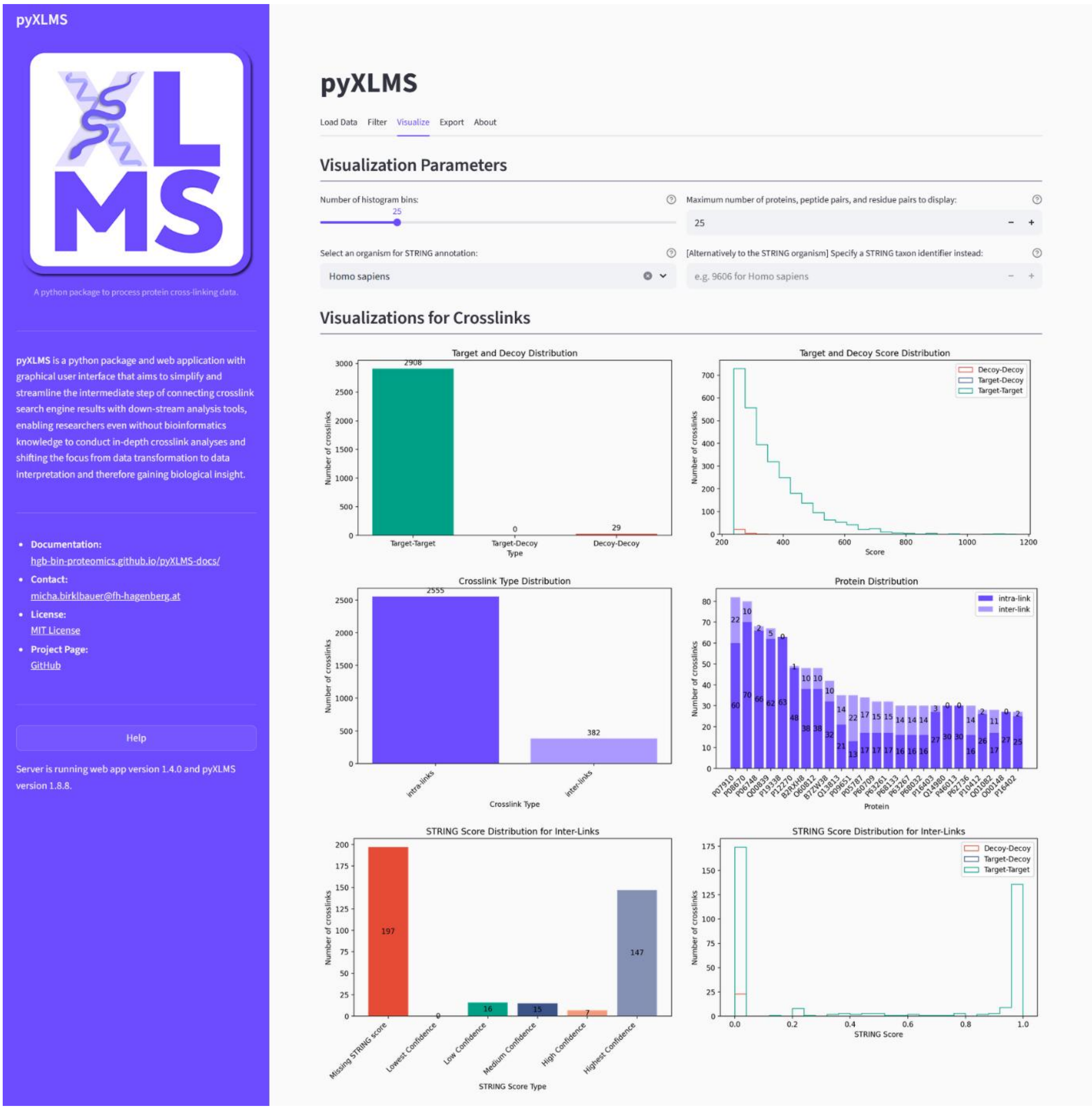

Supplementary Figure 2: *Overview of the visualization tab in the pyXLMS web application for crosslinks (XLs, in this case unique residue pairs). The first panel shows an overview of the target-target, target-decoy, and decoy-decoy XLs. The second panel shows the target-decoy score distribution. The third panel shows the number of intra- and inter-link XLs, while the fourth panel shows the number intra- and inter-link XLs per protein. Panel five shows the STRING [1] (main) score distribution for all interprotein XLs, sorted into the confidence classes suggested by STRING. In the sixth panel the STRING score is displayed as a distribution for target-target, target-decoy, and decoy-decoy XLs showing that decoy XLs all are assigned a zero STRING score which indicates that the protein-protein interaction does not exist in STRING. Visualizations are shown for results validated for 1% estimated residue pair-level FDR using data of human K562 nuclei from the same study as used in the main manuscript [2].*

This figure can be reproduced by uploading the file (search engine: Custom[1], crosslinker: DSBSO) to the web app (STRING organism "Homo sapiens" has to be selected in the Visualization tab):

https://github.com/hgb-bin-proteomics/pyXLMS-manuscript/blob/master/results/string/Nucleus_Rep1_Crosslinks.parquet

(g) Page 3: "We are also hosting a publicly available and free of charge web server that includes most functionality …". Does "includes most functionality" mean the standalone tool has more functionalities compared to the web server?

Yes, indeed. We missed to clarify this in the manuscript that there are several functions that are only available in the python package and not the web application. We have added information on that to the manuscript and additionally created a section in the Supplementary Information that outlines what exactly is not supported in the web application.

Actions taken:

Manuscript: Added clarification on which features are only supported in the python package, including a dedicated section in the Supplementary Information.

Software: We added a section called "Feature Support" to the documentation of the web app. We also referenced and clarified it in the newly added "FAQ" section of the user guide (available via this link: https://pyxlms.dev/docs/faq).

References:

*[1] Szklarczyk, D. et al. The string database in 2023: protein–protein association networks and functional enrichment analyses for any sequenced genome of interest. Nucleic Acids Research **51**, D638–D646 (2022).*
URL http://dx.doi.org/10.1093/nar/gkac1000.

*[2] Bräuer, P., Tirian, L., Müller, F., Mechtler, K. & Matzinger, M. In vivo crosslinking and effective 2D enrichment for proteome wide interactome studies. Communications Chemistry **8** (2025).*
URL http://dx.doi.org/10.1038/s42004-025-01644-6.

[1] The raw result file was created by MS Annika and can be found on PRIDE (PXD061173, filename: "Nucleus_Rep1.pdResult") but is too large to be processed in the public web app (1.3 GB). It can be processed with a local or self-hosted instance of the pyXLMS web app (further described here https://github.com/hgb-bin-proteomics/pyXLMS-app) or the pyXLMS python package (e.g. using:

```
pr = pyXLMS.parser.read(
        "Nucleus_Rep1.pdResult", engine="MS Annika", crosslinker="DSBSO", unsafe=True
)
```

). The "unsafe" parameter is required here because the result file contains erroneous crosslink positions, which is a bug with MS Annika. More can be read here:
https://hgb-bin-proteomics.github.io/pyXLMS/pyXLMS.parser.html#pyXLMS.parser.read_msannika
The linked "Nucleus_Rep1_Crosslinks.parquet" file simply contains the extracted crosslinks validated for 1% FDR and was then saved in parquet format to optimize the storage footprint. Processing of the raw result and exporting it to parquet took about ~20s where about half of it is spent on reading the raw result file (~81 000 unvalidated crosslinks and ~84 000 unvalidated CSMs).

Reviewer #3

(Remarks to the Author):

I co-reviewed this manuscript with one of the reviewers who provided the listed reports. This is part of the Nature Communications initiative to facilitate training in peer review and to provide appropriate recognition for Early Career Researchers who co-review manuscripts.

We thank the reviewer for their time and effort spent on reviewing our work and manuscript, and their valuable feedback!

Reviewer #4

(Remarks to the Author):

The authors present a python package and associated web interface that is able to take input from 7 different crosslink search algorithms. It can perform several basic tasks, including summarizing the data, filtering the data, calculating summary statistics and re-annotating crosslinks in cases where the identified peptides match multiple proteins. Importantly, pyXLMS is also able to output the data in formats that can then be read by and imported into several popular crosslink data analysis tools.
While the tool does perform several potentially useful functions many are already offered by existing tools. I do appreciate the author's point that if a researcher's XL analysis algorithm does not produce output compatible with the analysis tool they wish to use downstream then they need to convert their data. It is the reviewers opinion that this package could be useful to researchers wanting to convert crosslink data formats from the native output of the algorithm they used into a format suitable for use as input to one of these downstream analysis tools and I appreciate the effort made to cater for a wide range of inputs and outputs. I cannot recommend this work to be published in Nature Communications as it is not of sufficient novelty or impact. I think this work would be more suitable for submission as a brief technical note to a proteomics-specific journal (JPR, JASMS or similar).

We thank the reviewer for their time and effort reviewing our research and their overall positive assessment of our work!

We acknowledge that some functions in pyXLMS might already be covered by other tools, however we do also want to highlight that no other tool features such a broad support for different input sources and the sheer amount of features that we already have – which has grown already with this revision even beyond what was requested by reviewers and will further grow in the future. (E.g. see https://github.com/hgb-bin-proteomics/pyXLMS/releases, initial submission release was pyXLMS v1.7.5.)

In fact, pyXLMS is the first software package that provides this foundational layer to connect different crosslink search engines and input formats with data processing and quality control pipelines and ultimately a wide range of down-stream analysis tools. The creation of pyXLMS is a significant step forward in streamlining XL analysis and in increasing the accessibility of the field in itself, which arguably may be more impactful than a specialized methodological tool that only few people may be able to use.

In short, pyXLMS democratizes XLMS: Providing both a python package and public web application enables researchers without bioinformatics skills to perform in-depth XLMS analyses, broadens the user base, and therefore increases down-stream biological discoveries.

Our focus with pyXLMS has first and foremost been to create something that anyone can use, pyXLMS is not just the code or implementation, it is an extensive documentation of tools incorporating knowledge from countless discussions with other developers and clarifications on correct input and output formats. It relieves researchers from the responsibility of making sure that their data transformation scripts work correctly and avoids errors arising from misunderstandings or because of missing information. It will hopefully also increase reproducibility by decreasing the number of "inhouse scripts" that are never publicly shared but part of many publications focusing on other questions. Ultimately, pyXLMS is also a great resource

for learning crosslink data analysis, featuring a user guide with more than 50 pages of step-by-step instructions and highlighting many of the commonly used tools in the field.

***In short, FAIR + reproducible: The high level of interoperability in pyXLMS promotes reproducible, shareable workflows and aligns with FAIR data principles – an important (editorial) priority for high-impact journals such as* Nature Communications.**

Usability of pyXLMS is further reinforced by the fact that it is completely "AI ready". While we are personally rather cautious about generative AI usage, we recognize that it has become an important tool especially in code generation. The pyXLMS code base is not only fully documented with best practices, but our online user guide is fully markdown based and can be directly used as context in any AI assistant. Users can also directly interact with any page in the user guide seamlessly via the ChatGPT and Claude integrations. This effectively enables researchers who have never written a single line of python code to still use the python package, safeguarded by pyXLMS's fail fast philosophy that enforces that functions are not used with misinputs or incompatible data.

In short, open-source, extensible, and AI compatible: pyXLMS is highly accessible for community adoption, extension, and integration into other pipelines – qualities that increase long-term impact (and citation potential).

We have already had several positive interactions with people using pyXLMS (which were not involved in the project), some even publicly trackable on our GitHub (e.g. see https://github.com/hgb-bin-proteomics/pyXLMS/issues/201 and https://github.com/hgb-bin-proteomics/pyXLMS/issues/240), despite our work not being officially published yet. Publication in Nature Communications *would further amplify the visibility of our work and make people aware of this solution that will in our opinion inherently impact many groups working in the field of crosslinking.*

Moreover, XLMS data are used by structural biologists, cell biologists, and systems biologists (e.g. mapping interactomes, conformational changes, drug target studies). A tool that streamlines downstream XL-MS analysis therefore serves a wide, interdisciplinary readership appropriate for Nature Communications.

Last but not least Nature Communications *has been the venue of choice for impactful software as well as technical and benchmarking studies in the field of crosslinking mass spectrometry in the past (e.g. https://doi.org/10.1038/s41467-026-69489-8, https://doi.org/10.1038/s41467-025-64844-7, https://doi.org/10.1038/s41467-022-31701-w) and we therefore think that it is a perfect fit.*

Authors' Response Manuscript "Unified down-stream analysis of crosslinking mass spectrometry results with pyXLMS"

We want to thank the reviewers for taking the time and effort to review our revised manuscript and provide detailed feedback. We are again very grateful for the reviewers' comments which have substantially improved the quality of our work and this manuscript.

Below follows a point-by-point response to the comments made by the reviewers. The original reviewer comments are followed by our response in blue.

Text sections from the previous round of revision are indented and greyed out for better clarity.

Reviewers' comments:

Reviewer #1

(Remarks to the Author):

Reviewer's comments:

Thanks for reviewing the manuscript. Below I point some comments out.

> 1) Level of analysis: CSM/XL vs PPI
> The manuscript repeatedly states that pyXLMS focuses on CSM and XL aggregation and analysis.
> However, current XLMS applications place strong emphasis on PPI level questions (interactomes, network perturbation, ligand or toxin targeting, etc.), and this is also how the method is motivated in the abstract and introduction.
> • The current description does not clearly explain if or how CSM and XL information is systematically propagated to PPI level (e.g. how protein–protein interactions are defined, how ambiguous peptides are handled at PPI level, or how multi protein groups are treated).
> • FDR control at PPI level is not described, whereas FDR is discussed only for CSM and XL levels and only for a subset of engines (MaxLynx, MS Annika, xiSearch).
>
> In this context, I would suggest to:
> a) explicitly describe how pyXLMS derives PPIs from XLs: rules for grouping crosslinked residue pairs into protein–protein interactions, how ambiguity is handled, and how intra vs inter protein links are converted into network edges.
> b) clarify whether PPI level FDR is implemented, and if not, state this limitation clearly and discuss how users are expected to control error rates at PPI level (e.g. via external tools, recommended workflows, or future work).
> c) If PPI output is not supported, temper statements in the abstract and introduction regarding "identification of protein–protein interactions" to make clear that pyXLMS currently operates at CSM/XL level and relies on downstream tools to infer PPIs.
>
> *We apologize that this was not made clear enough in the manuscript, pyXLMS exclusively works on CSM and XL level and does not support PPIs directly. This decision was made because we currently do not support any down-stream analysis tools that would require PPIs as input (and have not encountered such a tool yet). Some of the down-stream analysis tools might output PPIs using the inputs generated by pyXLMS, but handling aggregation and validation is then happening on the side of the down-stream analysis tool.*

We might support PPI input in the future if we see enough use cases but currently have no plans to implement them. We have gone through the abstract and introduction again and PPIs are only mentioned as being applications of XLMS and not in the context of pyXLMS, we apologize if that came across wrong. We have added a statement in the pyXLMS description and in the limitations that explicitly clarifies that pyXLMS does not support PPIs directly at the moment.

Actions taken:

- ***Manuscript: We explicitly added that pyXLMS does not directly support PPIs as a limitation in both the pyXLMS description in the introduction, results, and in the discussion.***
- ***Software: We have added a FAQ (see https://pyxlms.dev/docs/faq) that specifically clarifies which aggregation levels pyXLMS supports and that PPIs are not supported.***

Thank for the explanation. However, it is important to note that, in contemporary XL-MS applications, the primary biological motivation is almost invariably the mapping of protein–protein interactions (PPIs) and interaction networks, rather than the isolated reporting of crosslinked residue pairs. Recent reviews explicitly position XL-MS as a tool for PPI discovery and characterization and for constructing dynamic interactomes at the systems level. Representative studies describe XL-MS–based efforts to define protein interaction landscapes in vivo, to perform in situ interaction proteomics and map extensive PPI maps in human cells and chromatin, and to leverage XL-MS for understanding endogenous PPI networks in the context of drug development against PPI modulators. Further recent work emphasizes XL-MS as a powerful approach for capturing protein network dynamics, from targeted mapping of PPIs to large-scale identification of interactome networks in cells and tissues. These examples illustrate that the field now routinely frames XL-MS as a PPI-centric technology, supporting the suggestion that the manuscript should more explicitly address how CSM/XL results are intended to be used for PPI-level questions and error control. Below you can find out some references that support the points above mentioned:

- Dynamic Interactomics by Cross-Linking Mass Spectrometry: Mapping the Daily Cell Life in Postgenomic Era – OMICS 2022.
    - Highlights XL-MS as a powerful tool for PPI discovery and characterization and for capturing dynamic interactomes.
- Chemical cross-linking and mass spectrometry enabled systems-level structural biology – Curr Opin Struct Biol 2024.
    - Emphasizes XL-MS for understanding protein–protein interaction networks relevant to physiology, pathology, and therapeutic targeting.
- Protein interaction landscapes revealed by advanced in vivo cross-linking–mass spectrometry – PNAS 2021.
    - Describes XL-MS applied to define protein interaction landscapes and PPIs in their native cellular environment.
- Optimized Cross-Linking Mass Spectrometry for in Situ Interaction Proteomics – J Proteome Res 2019.
    - Presents an XL-MS workflow for in situ interaction proteomics, mapping extensive protein–protein interaction maps in live human cells and chromatin.

The authors have provided clear and satisfactory responses to the remaining points raised in my review, adequately addressing the concerns about data processing description and the intended

user audience. In particular, their clarifications on implementation details and interoperability improve the transparency of the manuscript and bring it closer to the claims made in the abstract and introduction. However, it remains critically important to explicitly clarify the points raised regarding the level of analysis (CSM/XL vs PPI), as discussed in my previous comments. Given that the current XL-MS field overwhelmingly frames the technology as a PPI-centric tool for interactome and network analysis, leaving this aspect ambiguous risks misrepresenting the method's scope and limiting its interpretability for downstream users. Therefore, while I am satisfied with the authors' responses to the other questions, I strongly recommend that the revised manuscript include a clear statement on how (or whether) crosslinked residue pairs are propagated to PPI level, how ambiguity is handled at that level, and how error control is intended to operate for PPI-derived conclusions, or at least explicitly acknowledge these as limitations and outline a recommended workflow for users.

We thank the reviewer again for their time and effort as well as their thorough explanation and detailed feedback. We absolutely agree that the field of XL-MS has moved towards PPI-level analysis and how crucial aggregation and error control at that level are. The caveat for us is that most crosslink search engines either do not report PPI-level results or even worse, do not even report enough information to successfully aggregate and validate lower-level results up to PPI-level manually. We also do not want to reinvent the wheel and build a validation tool with pyXLMS when great algorithms/applications for specifically that purpose already exist, as for example xiFDR.

We have reconsidered implementing basic support for PPIs in pyXLMS but concluded that the added benefit in pyXLMS does – at least currently – not justify the effort that is required for properly integrating PPIs. More specifically, adding PPI support to pyXLMS would at the moment only unlock basic summary and filtering options for PPIs such as filtering by a certain protein or plotting a PPI score distribution. However, the PPI analysis would end after that in pyXLMS because there are currently no export options for PPIs and none of the supported down-stream analysis tools take PPIs as input. We have opened an issue in our GitHub repository regarding PPI support in pyXLMS for the community to give input and are happy to revisit this issue on demand. We do want to add PPI support to pyXLMS in the future, especially if more crosslink search engines start to support it and more down-stream analysis tools are built specifically for PPIs, but it is not a goal at this moment or for this manuscript.

We currently list the limitation that pyXLMS does not support PPIs at three different locations in the manuscript.

- *First in the introduction, page 3: "pyXLMS focuses on data processing at the CSM and XL level with PPIs only being indirectly supported and derived via export to other down-stream analysis tools."*
- *Secondly, in the results, page 4: "It should be explicitly noted here that pyXLMS focuses on reading, processing, and exporting data on CSM and XL aggregation levels and does not support protein-protein interaction (PPI) level data, neither in reading nor processing. This has been an intentional design decision as the number of crosslink search engines supporting PPI level results is still limited and currently none of the down-stream analysis tools integrated with pyXLMS require PPI input."*
- *And lastly, in the discussion, page 13: "Additionally, pyXLMS focuses on reading, processing, and exporting data on CSM and XL aggregation levels and does not support PPI level data, neither in reading nor processing. This has been an intentional design choice as the number of crosslink search engines supporting PPI level results is still*

limited and currently none of the down-stream analysis tools integrated with pyXLMS require PPI input."

We have extended the discussion now with some additional suggestions that we would recommend users who are particularly interested in PPIs, e.g. we added the following text:

"We recommend users who are particularly interested in PPI-level analysis to preferably use a crosslink search engine that does support PPI level results (e.g. Scout) or that supports validation via xiFDR (e.g. xiSearch, MS Annika) which can handle aggregation and validation up to the PPI level. Results at the PPI level can then be easily further analyzed in tools like Cytoscape. Generally, pyXLMS can transform lower-level results for usage with xiFDR to support this process, or alternatively export them to xiVIEW which can also aggregate CSMs and XLs to PPI networks."

We are also happy to rephrase any of the text passages in our manuscript if the reviewer has any suggestions or preferences on how to maybe even better clarify these points!

Actions taken:

Manuscript: We have extended the discussion by adding recommendations for users specifically interested in PPI-level analysis.

Reviewer #1

(Remarks on code availability):

The Python code provided adheres to the standard rules of Python syntax and semantics: it uses valid identifiers, correct indentation, proper punctuation (parentheses, brackets, and commas), and follows the language's statement and expression structure. All imports, function definitions, loops, conditionals, and data manipulations are written in a way that would execute without syntax errors in a standard Python environment. The logic is clear, the variable names are meaningful, and the code is organized in a readable, maintainable form, making it satisfactory for the intended purpose.

We thank the reviewer for thoroughly reviewing and approving our code!

Reviewer #4

(Remarks to the Author):

My concerns have been rebutted in the revisions.

We thank the reviewer again for their time and effort spent reviewing our work and manuscript!

The manuscript "Unified down-stream analysis of crosslinking mass spectrometry results with pyXLMS" presents a useful and timely framework to make intuitive the analysis across different search engines. The description of the data processing is clear and informative, and the algorithm is said to speed up the post-analysis not only for bioinformaticians but also for non-bioinformaticians. However, important aspects of the post-analysis, interoperability, and implementation details need clarification or correction to match the claims made in the text and abstract.

1) Level of analysis: CSM/XL vs PPI

The manuscript repeatedly states that pyXLMS focuses on CSM and XL aggregation and analysis. However, current XLMS applications place strong emphasis on PPI-level questions (interactomes, network perturbation, ligand or toxin targeting, etc.), and this is also how the method is motivated in the abstract and introduction.

- The current description does not clearly explain if or how CSM and XL information is systematically propagated to PPI level (e.g. how protein–protein interactions are defined, how ambiguous peptides are handled at PPI level, or how multi-protein groups are treated).
- FDR control at PPI level is not described, whereas FDR is discussed only for CSM and XL levels and only for a subset of engines (MaxLynx, MS Annika, xiSearch).

In this context, I would suggest to:

a) explicitly describe how pyXLMS derives PPIs from XLs: rules for grouping crosslinked residue pairs into protein–protein interactions, how ambiguity is handled, and how intra- vs inter-protein links are converted into network edges.
b) Clarify whether PPI-level FDR is implemented, and if not, state this limitation clearly and discuss how users are expected to control error rates at PPI level (e.g. via external tools, recommended workflows, or future work).
c) If PPI output is not supported, temper statements in the abstract and introduction regarding “identification of protein–protein interactions” to make clear that pyXLMS currently operates at CSM/XL level and relies on downstream tools to infer PPIs.

2) mzIdentML adoption and positioning

The introduction claims that mzIdentML 1.2/1.3 has “not seen wide adoption in the field of XLMS with only a handful of crosslink search engines and down-stream analysis tools supporting the format.” At the same time, you list mzIdentML 1.2 support in pyXLMS and emphasize interoperability as a main contribution. Given that many current engines do export mzIdentML 1.2/1.3, this statement feels outdated and undersells the existing ecosystem.

In this regard please:

a) Re-evaluate and soften the statement on “not seen wide adoption,” or back it up with an up-to-date, quantitative overview of which crosslink engines and viewers support mzIdentML 1.2/1.3.
b) Highlight that pyXLMS can act as a bridge layer on top of the emerging mzIdentML 1.3 crosslink support, rather than implying the standard is rarely used.
c) Consider recommending mzIdentML 1.2/1.3 as a default interchange format for XLMS, with pyXLMS adding convenience and export functions on top of it.

3) FDR and engine coverage

The manuscript states that FDR estimation in pyXLMS is “currently only the case for MaxLynx, MS Annika, and xiSearch,” because these provide targets/decoys and a single score. However, Scout is also described as providing CSV output and is included among the supported engines, and you mention reading both CSM and XL levels for Scout.
In the current XLMS landscape, Scout can produce target/decoy information at CSM, XL, and PPI levels, and it would be important to clarify if and why this is (not) used in pyXLMS. If technical reasons prevent using Scout’s outputs for FDR, explain the limitation explicitly (e.g. missing combined score, multiple scoring dimensions, or format specifics). In addition, discuss whether PPI-level FDR is planned, in particular leveraging engines like Scout that provide decoy PPIs.

4) Intersection of multiple engines in the web app

The manuscript claims that pyXLMS “allows to easily create intersections of CSMs and XLs, either from different replicates or different crosslink search engines.” In the web application description, users are guided through upload, filtering, visualization, and export.
In practice, when using the public web app, the upload step enforces selection of a single engine/format from a dropdown, making it unclear how intersections across different search engines can be created in the GUI workflow you advertise for non-programmers.

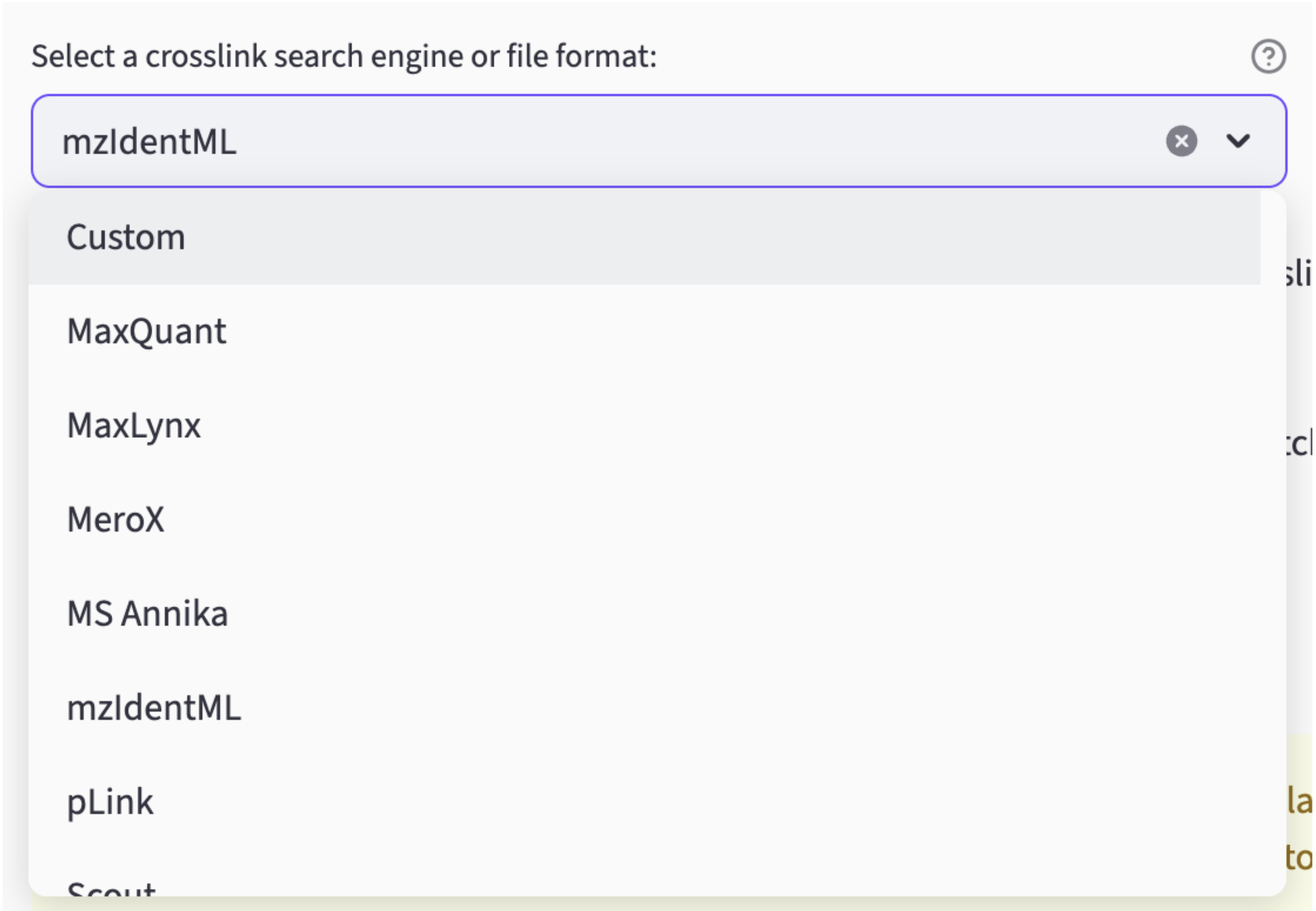

In this context, I would suggest clarifying whether multi-engine intersections are currently only available in the Python API, and if so, state this explicitly and adjust the claims about the web app accordingly. Additionally, provide a brief and explicit example (user interface steps or code) demonstrating how to obtain the intersections of XLs in pLink, Scout, and XlinkX for the Cas9 dataset.

5) **Export options vs Table 2 (xiView, MS Annika)**

Table 2 lists xiVIEW as a supported export format (XL only) and does not mention MS Annika as an export target. The text emphasizes “more than ten different down-stream analysis tools and formats” and cites xiVIEW as a primary example. In the public web app, however, the export options presented to the user include only IPM-X-FDR, MS Annika, ProXL and xiFDR, with xiVIEW not available and MS Annika not listed in Table 2.

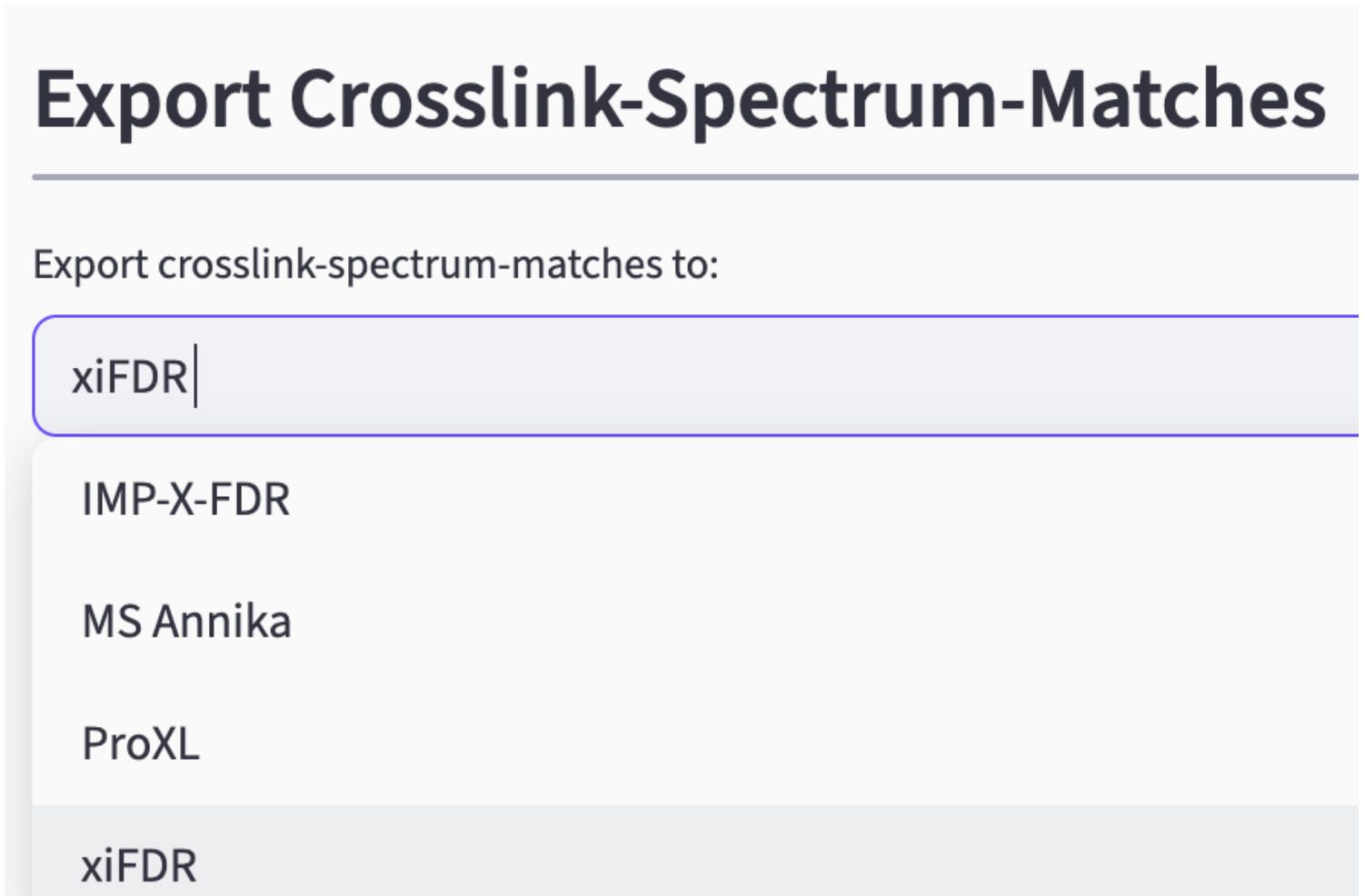

So, I would suggest reconciling Table 2, the main text, and the web app implementation. Ensure that xiVIEW export is consistently available in the web app if claimed in the manuscript or explicitly mark it as “currently only via Python API” if that is the case. In addition, add a note in the Methods or Implementation section stating that the set of export formats in the hosted web app may lag behind the Python package and specify what is guaranteed at the time of publication.

6) Versioning and Scout choice for the benchmark

Section 4.1 states that you tested Scout version 1.6.3 (Borges repository) and Scout 2.0.0 beta (Liu Lab repository). In the data analysis section, you report using Scout 2.0.0 beta from the Liu Lab for the Cas9 benchmark. For many users, the stable Scout 1.6.3 (or 2.0.0) from the Borges repository is now a practical default and, in some datasets, performs better than the beta variant.

In this case, justify the exclusive use of the beta 2.0.0 version in the main analysis; at minimum, mention that alternative releases (e.g. Scout 2.0.0 from Borges) exist and might produce different or improved results.

Moreover, if feasible, re-run the Cas9 dataset with the stable Scout 2.0.0 (Borges) and report CSM, XL, and PPI numbers at 1% FDR alongside the existing pLink and XlinkX results. If re-analysis is not possible, explicitly discuss how sensitive your conclusions (e.g. relative performance of pLink vs Scout) might be to the specific Scout version used.

7) Installation requirements and reproducibility

The Implementation section details Python packages and testing (pandas, pyteomics, biopython, matplotlib, biopandas, lxml, streamlit, sphinx, etc.) and reports 584 tests

covering parsing and core functionality. However, minimal system requirements are not stated explicitly.

However, there are a lack information about the package versions used during the development as well as the minimum set up for the machine. In this context, add a short subsection (e.g. “System requirements”) specifying at least: minimum Python version (e.g. 3.9+), required operating systems (Linux, macOS, Windows) and tested versions, approximate RAM/disk requirements for typical XLMS datasets, and any known performance constraints.

On the other side, for the web app, clarify what is needed to run locally or self-host (Python version, streamlit version, browser compatibility). Since the authors emphasized non-expert usability, make sure the user guide (cited URLs) includes a minimal “one-page” install recipe that matches the versions used in this manuscript.

Minor comments and suggestions

1. **Consistency in engine and format lists**

The text at multiple points counts “seven different crosslink search engines” but then lists MaxLynx, MeroX, MS Annika, pLink 2/3, Scout, xiSearch/xiFDR, and XlinkX, which reads as more than seven logical tools depending on how they are grouped. For clarity, define whether xiFDR and XlinkX are treated as separate input engines or as auxiliary formats.

2. **Clarify the mzIdentML version support**

The authors explicitly mention support for mzIdentML 1.2 in the tests, and cite mzIdentML 1.3 as a format that extends crosslink reporting. It would be helpful to state clearly whether 1.3 is fully supported as input (and tested) or treated as 1.2 with partial coverage.

3. **Documenting limitations more prominently**

The Discussion already notes that lack of decoys in some engines prevents validation and export to xiFDR and that varying information content across engines limits some exports. It would help readers if the web app and docs expose these limitations explicitly (e.g. when a user uploads MeroX results and tries to export to xiFDR, a clear message that FDR cannot be recomputed in pyXLMS).

4. **Terminology**

Ensure consistent use of terms like “crosslink/residue pair,” “XL,” and “PPI” throughout the text and figures and clarify early on that the core data structures operate at CSM and XL levels, with PPIs derived rather than directly stored.
